# Neq2X7: a multi-purpose and open-source fusion DNA polymerase for advanced DNA engineering and diagnostics PCR

**DOI:** 10.1186/s12896-024-00844-7

**Published:** 2024-04-02

**Authors:** Cristina Hernández-Rollán, Anja K. Ehrmann, Arsenios Vlassis, Vijayalakshmi Kandasamy, Morten H. H. Nørholm

**Affiliations:** 1 The Novo Nordisk Foundation Center for Biosustainability, Technical University of Denmark, Søltofts Plads, Building 220, Kongens Lyngby, 2800 Denmark; 2 Mycropt ApS, Kongens Lyngby, 2800 Denmark

**Keywords:** *Nanoarchaeum equitans*, Neq DNA polymerase, Polymerase chain reaction, DNA polymerase fidelity, PCR inhibitors, Modified nucleotides, Uracil-excision cloning, USER cloning, dUTP

## Abstract

Thermostable DNA polymerases, such as Taq isolated from the thermophilic bacterium *Thermus aquaticus*, enable one-pot exponential DNA amplification known as polymerase chain reaction (PCR). However, properties other than thermostability - such as fidelity, processivity, and compatibility with modified nucleotides - are important in contemporary molecular biology applications. Here, we describe the engineering and characterization of a fusion between a DNA polymerase identified in the marine archaea *Nanoarchaeum equitans* and a DNA binding domain from the thermophile *Sulfolobus solfataricus*. The fusion creates a highly active enzyme, Neq2X7, capable of amplifying long and GC-rich DNA, unaffected by replacing dTTP with dUTP in PCR, and tolerant to various known PCR inhibitors. This makes it an attractive DNA polymerase for use, e.g., with uracil excision (USER) DNA assembly and for contamination-free diagnostics. Using a magnification via nucleotide imbalance fidelity assay, Neq2X7 was estimated to have an error rate lower than 2 ∙ 10^−5^ bp^−1^ and an approximately 100x lower fidelity than the parental variant Neq2X, indicating a trade-off between fidelity and processivity – an observation that may be of importance for similarly engineered DNA polymerases. Neq2X7 is easy to produce for routine application in any molecular biology laboratory, and the expression plasmid is made freely available.

**Supplementary Information:**

The online version contains supplementary material available at 10.1186/s12896-024-00844-7.

## Introduction

Exponential DNA amplification by the polymerase chain reaction (PCR) is one of the most important inventions in molecular biology, and the technique was paradigm-shifting in DNA diagnostics and forensic science [1]. DNA polymerases are the central enzymes in PCR and are at the frontier of other biotechnological applications [2, 3]. Many contemporary synthetic biology methods rely entirely on PCR, and DNA polymerase properties like high fidelity and processivity are imperative. Applications that require exceptional DNA polymerases include advanced DNA assembly [4–10], medical diagnostics [11], and technologies that require the incorporation of non-conventional nucleotides such as xeno-nucleic acids (XNA) [12]. Additionally, next-generation DNA sequencing approaches are continuously refined and rely on DNA polymerases that tolerate specifically labeled substrates [13]. As a final point, routine PCR workflows that allow for DNA amplification in short time frames - as low as 3 min [14] - are crucial for rapid detection of, e.g. pathogens, and are particularly relevant during pandemic events, such as the recent COVID-19 crisis.

Most PCR methods rely on the action of a thermostable DNA polymerase with high processivity and low error rate [15]. Depending on the specific application, other desired features include high DNA yield, and amplification of long (long-range PCR) or complex templates (e.g., DNA with high GC content or secondary structure). In specific cases, it is a requirement that the DNA polymerase tolerates unconventional nucleotides such as deoxyuridine triphosphate (dUTP). The popular DNA assembly method USER (Uracil-Specific Excision Reagent) cloning [6, 16, 17] utilizes uracil bases in the PCR primers that are subsequently excised to create short, compatible single-stranded overhangs. Incorporation of dUTP into the DNA template can also limit carry-over of amplification products in sensitive environments such as forensic laboratories [18]; when PCRs are performed using dUTP instead of dTTP, reactions can be treated with uracil-N-glycosylase (UNG) before amplification. This way, uracil-containing DNA is degraded, and only DNA in the new test sample is left intact [19]. Uracil-accepting polymerases should also amplify environmental samples more robustly since some uracil occurs naturally in DNA [20].

DNA polymerases originating from thermophilic and hyperthermophilic archaea, mainly belonging to family B, are ideal candidates for PCR, as they share most of the crucial characteristics for advanced applications: they are thermostable and show high-fidelity thanks to their proofreading (3’-5’-exonuclease) activity [21]. Furthermore, they can amplify long stretches of DNA and, in contrast to the widely used Taq polymerase, do not add an extra dA at the 3’ end. These characteristics make them highly attractive for PCR and molecular cloning techniques. Still, a wild-type DNA polymerase rarely possesses all the ideal characteristics needed, and protein engineering is often carried out to improve the performance [13].

In 2006, the expression and characterization of a new archaeal DNA polymerase from the hyperthermophile *Nanoarchaeum equitans* (Neq) was reported [22]. In follow-up work, the use of Neq for PCR was described and it was discovered that, in contrast to other archaeal family B DNA polymerases, Neq does not stall when encountering uracil, as it lacks the specific binding pocket [23, 24]. Additionally, it was found that Neq has high processivity but low fidelity. However, the fidelity was improved through the incorporation of two mutations (A523R/N540R) which brought the error rate of the Neq polymerase *on par* with the highly popular Pfu polymerase [25]. Nevertheless, despite its apparent attractive properties as a high-performance polymerase for in vitro applications, Neq does not yet seem to be widely used as judged by a simple internet keyword search (Supplementary Fig. 1).

There are different ways to engineer a DNA polymerase for enhanced processivity. One of the simplest approaches reported to date is the attachment of an unspecific DNA binding domain [26]. The DNA binding domain of *Sulfolobus solfataricus* (known as Sso7d domain) enables higher processivity through its dsDNA binding ability without significantly changing the other catalytic properties of the polymerase [3, 7]. Here we engineer and characterize a new variant of the Neq polymerase, Neq2X7, that combines the two fidelity-increasing mutations (2X) previously reported with the addition of Sso7d [26, 27]. Neq2X7 is easily produced with a routine protein production workflow and the construct is freely shared with the community and accessible via the Addgene repository.

## Results and discussion

### Expression plasmid construction, DNA polymerase expression, and purification

**Fig. 1:**
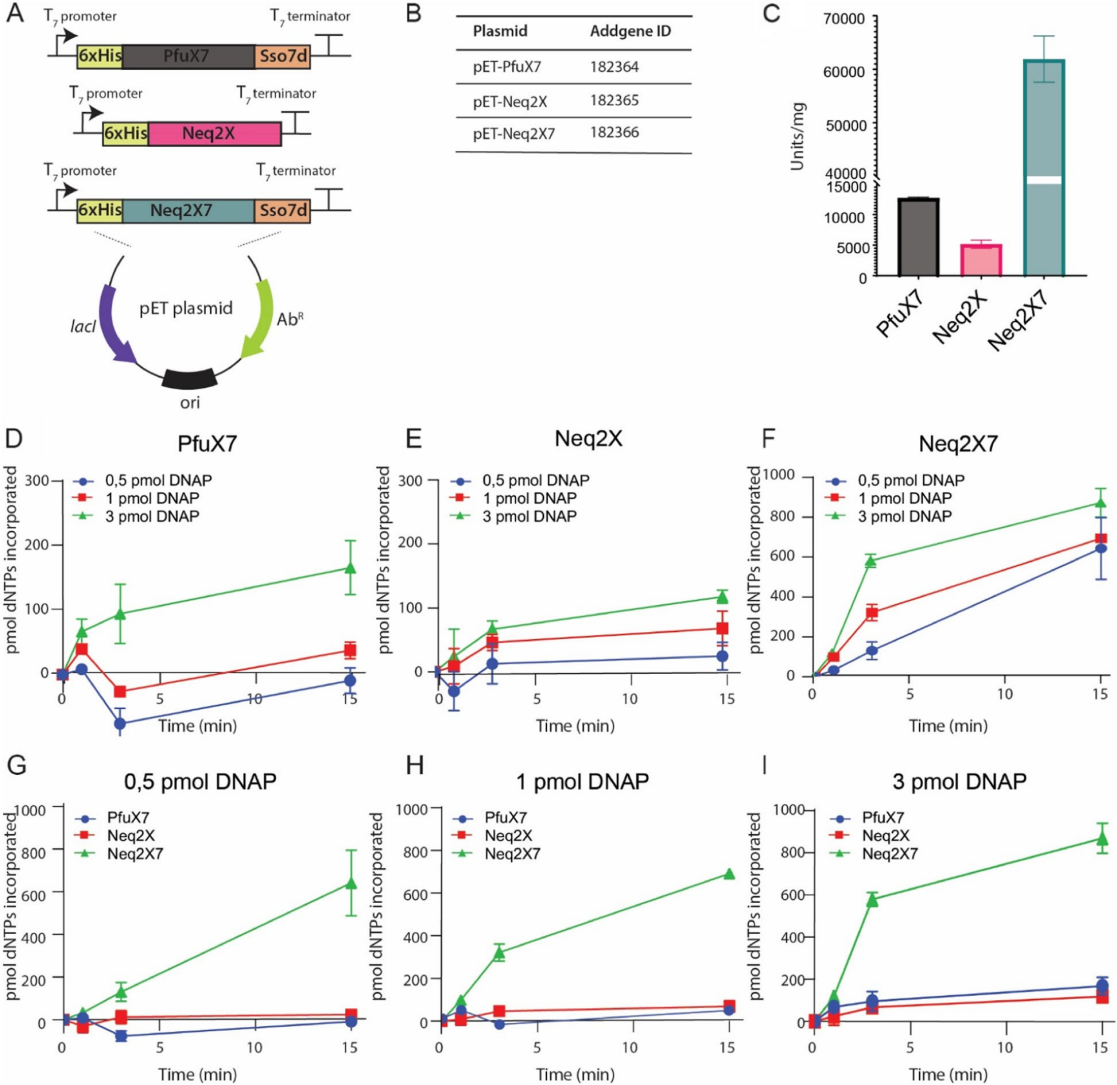
Illustration of the plasmids used in this study and comparison of the PCR extension rates of the DNA polymerases PfuX7, Neq2X, and Neq2X7 measured by binding of the fluorescent Pico488 dye to double-stranded DNA (**A**) Schematic overview of the PfuX7, Neq2X, and Neq2X7 expression plasmids. **B** Table with plasmids used in this study, including their Addgene IDs. **C** DNA polymerase activity units calculated based on incorporation of dNTPs in 10 min per mg of purified enzyme. **D** Fluorescence-based activity assay for PfuX7, (**E**) Neq2X, and (**F**) Neq2X7 using 0.5, 1, and 3 pmol of enzyme over time. **G** Fluorescence-based activity assay comparing dNTP incorporation rates between PfuX7, Neq2X, and Neq2X7 for 0.5 pmol, (**H**) 1 pmol, and (**I**) 3 pmol of each enzyme and over time. All measurements were performed in triplicates, and the graph displays the median with the standard deviation indicated. DNAP (DNA Polymerase). Pico488 measurements were done using a Synergy H1 plate reader (BioTek Instruments, Inc)

The double mutant (A523R/N540R) Neq2X gene was ordered as a synthetic gene and cloned with or without the Sso7d-encoding domain into two different pET plasmids for production in *Escherichia coli* Rosetta2 (DE3) in combination with the pLysS plasmid as previously described for Pfu [7]. The resulting DNA polymerases carry 6xHis purification tags at their N-termini, and Neq2X7 harbors the Sso7d DNA fusion domain at the C-terminus (Fig. 1A, Supplementary Note S1). The C-terminus was chosen because the N-terminus is involved in binding template DNA [28]. Using a standard protein production protocol (see methods and Supplementary Fig. 2), we estimate that we produce enough Neq2X7 DNA polymerase for about 50,000 PCR reactions from 100 mL of bacterial batch culture. The constructs are summarized in a table in Fig. 1B along with Addgene repository accession numbers [29].

### Benchmarking the activity of Neq2X7 with similar DNA polymerases

The performance of Neq2X7 was compared with the double mutant Neq polymerase without the Sso7d DNA binding domain (Neq2X) and the Pfu-derived Sso7D fusion DNA polymerase PfuX7 [7] using a fluorescence-based DNA polymerase assay. PfuX7 is based on a mutant of the Pfu polymerase with diminished uracil binding affinity [7, 24]. This analysis showed a high activity of Neq2X7 compared to the other two polymerases, as reflected in the measured units per mg of the enzyme (Fig. 1C). The activity was extrapolated from the initial time points at which the rate of incorporation of dNTPs progresses in a linear fashion (Fig. 1D-I) and was normalized by the amount of enzyme. Using three different protein concentrations (0.5, 1, and 3 pmol) and time points at 1, 3, and 15 min, it can be observed that Neq2X7 incorporates more dNTPs on shorter timescales, even with less enzyme present. While polymerase activity is detected with as low as 0.5 pmol Neq2X7 (Fig. 1F), PfuX7 and Neq2X only show detectable nucleotide incorporation at 3 pmol (Fig. 1D and E). Units are typical measures of enzyme activity and are defined here as the amount of polymerase that incorporates ten pmol of dNTPs using a primer together with single-stranded viral DNA from M13mp18 as a template at 72 °C in 10 min. The activity of the Neq2X7 increases about eight-fold with the addition of the Sso7d DNA binding domain.

**Fig. 2:**
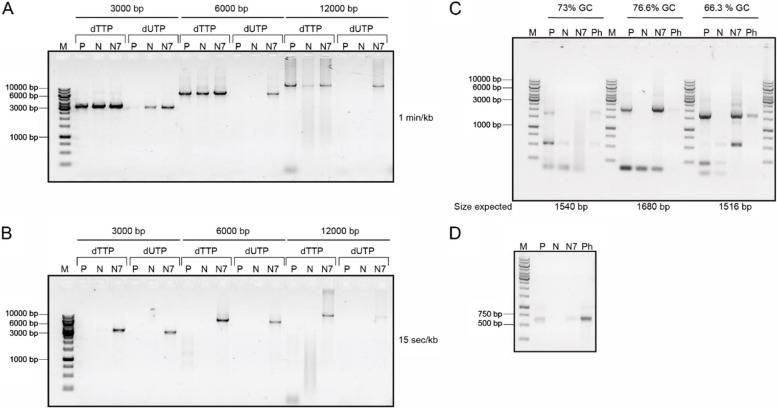
PCR comparison of the DNA polymerases performance for different templates, amplification times, and conditions using 1 pmol of each DNA polymerase enzyme run on a 1% agarose gel. **A** Three different amplicons with sizes 3000, 6000, and 12,000 bp were produced using 1 min of elongation per kilobase. For each template, two conditions were evaluated: (i) the amplification of DNA with a mixture of dNTPs containing dTTP (dATP, dTTP, dCTP, and dGTP), and (ii) dUTP replacing dTTP (dATP, dUTP, dCTP, and dGTP). **B** The same templates as in panel A were amplified using 15 s of elongation time per kilobase. **C** High GC content template amplification from three different genomic DNA regions of *Streptomyces* were amplified by PCR. From left to right, 73% GC of 1540 bp expected size, 76.6% GC of 1680 bp expected size, and 66.3% GC of 1516 bp expected size. **D** Fast-PCR amplification of a 630 bp template. P (PfuX7), N (Neq2X), N7 (Neq2X7), and Ph (Phusion) DNA polymerases. (M) DNA marker in base pairs. For PCR conditions, primers, and templates, see the Methods section

### Benchmarking the performance of Neq2X7 in various PCR applications

One classical challenge of PCR is the amplification of very long DNA stretches since it increases the risk of premature replication termination and therefore highly processive DNA polymerases are desired [21]. We compared Neq2X7, Neq2X, and PfuX7 PCR performance using three different amplicon sizes: 3, 6, and 12 kb. With an extension time of 1 min/kb at 72 °C in the PCR program, all three polymerases amplified DNA to a detectable level (Fig. 2A). However, when the extension time was shortened to 15 s/kb, only Neq2X7 was able to produce the desired DNA fragments (Fig. 2B). This indicates that the processivity of Neq2X7 is high compared to Neq2X and PfuX7.

A normal characteristic of archaeal DNA polymerases is the presence of a conserved domain that binds to uracil and stalls DNA amplification [21]. It is believed that this domain evolved to protect some archaeal organisms from the mutagenic incorporation of an adenine nucleotide as a result of deamination of cytosine into uracil on the opposite strand. However, the Neq polymerase lacks this uracil-binding pocket and therefore has the natural ability to tolerate uracil in the template and PCR amplification with dUTP nucleotides. In contrast, the “X” in PfuX7 denotes an engineered mutation that compromises the uracil-binding activity of this polymerase. We compared the performance of Neq2X7, Neq2X, and PfuX7 in PCR amplification with dUTP entirely replacing dTTP in the dNTP mix. For the shorter amplicons of 3000 bp, all tested polymerases were able to generate a detectable PCR product with an extension time of 1 min/kb (Fig. 2A). For longer amplicons, and at the reduced extension time of 15 s/kb, only Neq2X7 was able to amplify the target DNA successfully with dUTP (Fig. 2B). This indicates that Neq2X7 is a superior polymerase for applications requiring dUTP such as for limiting template contamination or USER cloning.

Often, a limitation in the use of DNA polymerases in PCR is the amplification of DNA with high GC content [30]. Many biotechnologically interesting organisms, such as *Streptomyces* and mycobacteria [31, 32] – and sometimes specific genomic regions [33] – have high GC content and therefore are challenging to work with. We evaluated the performance of Neq2X7 on high GC content templates using three different templates from genomic DNA isolated from *Streptomyces* ranging from 66 to 76% GC (see Supplementary Table 4). While Neq2X was unable to amplify any of the targets, the correct product was detected for Neq2X7 in two out of three cases (Fig. 2C). PfuX7 and the commercially available Phusion DNA polymerase were able to amplify the correct product for all three reactions. Thus, the Pfu polymerases may be better choices for high GC content DNA.

Fast DNA amplification is important for diagnostics and allows for amplification of DNA targets in the range of minutes. Inspired by the promising results on the high processivity of Neq2X7, we wanted to test whether this improved polymerase can perform “Fast-PCR”, and how it would compare to other highly processive polymerases (e.g., PfuX7 and Phusion). A 653 bp DNA fragment was selected for amplification using a fast-PCR program with a total runtime of 24 min (Fig. 2D and Supplementary Table 5). Both PfuX7 and Neq2X7 show a detectable signal on the agarose gel after the PCR, even though they are outperformed by the commercially obtained Phusion polymerase. Further optimization of the protocol and the buffer conditions, as well as utilization of specific Fast-PCR equipment, might enable Neq2X7 to compete head-to-head with the fastest PCR protocols currently available.

A common strategy to increase the efficiency of PCR reactions is to mix Taq polymerase with archaeal family B DNA polymerases. This strategy was previously employed to enhance the performance of the Neq polymerase [23]. We tested the combination of Neq2X7 and the commercial DreamTaq in two different concentrations to amplify two different targets (Supplementary Fig. 3) but did not observe considerable improvements in DNA amplification. Instead, there is an increase in unspecific amplification products if Neq2X7 is either mixed with DreamTaq, or if the concentration of Neq2X7 is increased. DreamTaq alone was not able to amplify the targets in the applied reaction conditions. These results highlight that adding more DNA polymerase does not always result in better results, and that mixtures of different polymerases should be balanced well to achieve the desired outcomes. Other polymerase combinations or further optimization of the reaction conditions could be interesting to test with Neq2X7.

### Evaluation of Neq2X7 fidelity

The introduction of two mutations in the Neq polymerase simultaneously improved processivity and fidelity [25]. In this study we show that the processivity is further increased by adding the Sso7d domain. While Wang et al. reported that the addition of the Sso7d domain did not impact Pfu DNA polymerase fidelity [26], other studies have suggested potential tradeoffs between base substitution fidelity and processivity [34, 35]. We used the Magnification via Nucleotide Imbalance Fidelity assay (MagNIFI) [36] to estimate the fidelity of the Neq2X7 polymerase as compared to Neq2X and the commercially available Taq and Phusion DNA polymerases.

**Fig. 3:**
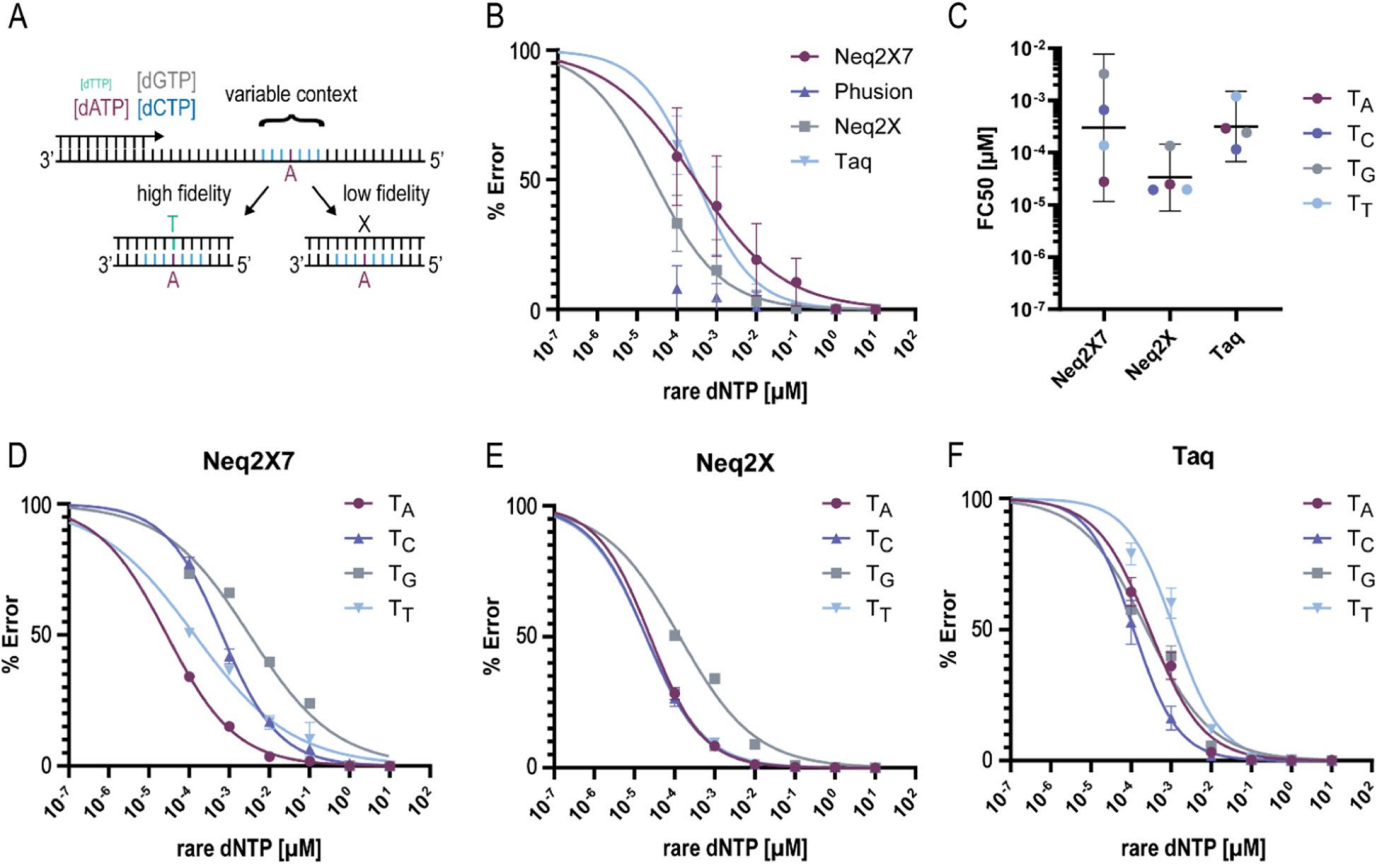
Determination of Neq2X7 fidelity (**A**) Schematic of the MagNIFI assay principle inspired by de Paz et al. **B** Frequency of mismatches observed at the error enrichment site for all four polymerases, measured across four different templates (*n* = 8). A nonlinear response curve was fitted to the data obtained from Neq2X, Neq2X7, and Taq samples. **C** Concentration of rare dNTP resulting in a 50% error frequency (FC_50_) obtained from a nonlinear fit to the error rate measurements based on the four individual templates. The geometric mean with a 95% confidence interval is shown underlying the individual data points. **D**, **E** Error frequency measurements and response curve fit resolved by the individual templates for Neq2X7, Neq2X, and Taq. Data points represent the mean, the error bars show the standard deviation of two biological replicates

The MagNIFI assay can determine single nucleotide variant error rates using Illumina DNA sequencing while limiting the number of bases that need to be sequenced for an accurate measurement. The assay is based on a primer extension reaction of a synthetic ssDNA template lacking one of the four nucleobases except at one specific position, the error enrichment site. By limiting the availability of the compatible nucleotide, the number of misincorporations at the error enrichment site is increased beyond the native DNA polymerase error rate (Fig. 3A). To exclude bias of misincorporation for a particular sequence context, four different templates are used, each with a different base at the error enrichment site and a variable sequence context library spanning the six nucleotides surrounding the error enrichment site (Supplementary Table 6). It was previously shown that the FC_50_ of a response curve fit to measurements of the error frequency obtained with different concentrations of the rare dNTP is a metric that correlates with the native error rate of the DNA polymerase [36].

Figure 3B shows the rate of misincorporation at the error enrichment site measured for the four different polymerases on four different extension templates. The number of errors incorporated by Phusion polymerase is very low across all rare dNTP concentrations, and therefore, no response curve was fitted to this dataset. None of the analyzed samples reached an error rate above 80%, even with a rare dNTP concentration as low as 10^−4^ µM. The data still allowed for a reasonable fit of response curves and determination of the FC_50_ metric. Of the other three polymerases, we obtained the lowest FC_50_ for Neq2X (2.6 ∙ 10^−5^ µM, Fig. 3C). In comparison, the FC_50_ values were increased by one order of magnitude for Neq2X7 and Taq (2.9 ∙ 10^−4^ µM, 3.0 ∙ 10^−4^ µM). However, a closer evaluation of the data separated by the four different templates revealed a much lower FC_50_ metric for Neq2X7 on A and T-based error enrichment sites compared to G and C positions (Fig. 3D). Neq2X also shows a higher error frequency for the G template, while we did not observe any clear difference between the different extension templates for Taq polymerase (Fig. 3E, F). We analyzed the base substitution profile at the error enrichment site for Neq2X7, Neq2X, and Taq and found no specific differences between the three polymerases (Supplementary Fig. 4). The observed error profiles align with those reported previously [36].

The obtained FC_50_ values are outside the range for which de Paz et al. calibrated a conversion formula of the FC_50_ metric into polymerase error rates. Therefore, we cannot confidently perform an accurate conversion but rather report estimates of the error rate, which are given in Table 1. Based on a comparison with the error rate for Taq polymerase reported by the manufacturer, we can assume that our measurements underestimate the error rate by at least a factor of 10 (Table 1). Even when taking this correction into account, we measured a much lower error rate for Neq2X than Ppyun et al. previously reported [25]. This could be due to the different experimental approaches. The MagNIFI assay can only resolve single nucleotide substitutions and indels. The *lacZ*-based mutation assay can detect a broader spectrum of different mutations but is, in contrast, blind to silent nucleotide substitutions and is limited in terms of the available sequence context. Aside from the evaluation of the errors at the error enrichment site, we did observe a decrease in high-quality reads obtained from extension reactions performed with Taq polymerase at low concentrations of the rare dNTP (Supplementary Figs. 5 and 6). These changes in fragment size could indicate other types of errors (e.g., larger deletions) that our analysis pipeline cannot capture. We made similar observations for extension reactions obtained with Phusion polymerase on A and C templates (Supplementary Fig. 5).

**Table 1:** Fidelity metrics and estimated error rates for Neq2X and Neq2X7

	FC_50_ all templates [μM]	Error rate estimated according to de Paz [bp^-1^]	Literature / Manufacturer reference [bp^-1^]
Neq2X	2.6×10^-5^	1.3×10^-8^	6.3×10^-5^ [25]
Neq2X7	2.9×10^-4^	1.8×10^-6^	
Taq	3.0×10^-4^	2.0×10^-6^	2.2×10^-5^ [37]
Phusion			4.4×10^-7^ [38]
Error rate improvement compared to Taq			
Template	Neq2X	Neq2X7	
All templates	155	1.1	
T_A_	177	139	
T_T_	285	5.2	
T_C_	289	0.2	
T_G_	5.5	0.01	

### Assessment of Neq2X7 performance in the presence of common PCR inhibitors

**Fig. 4:**
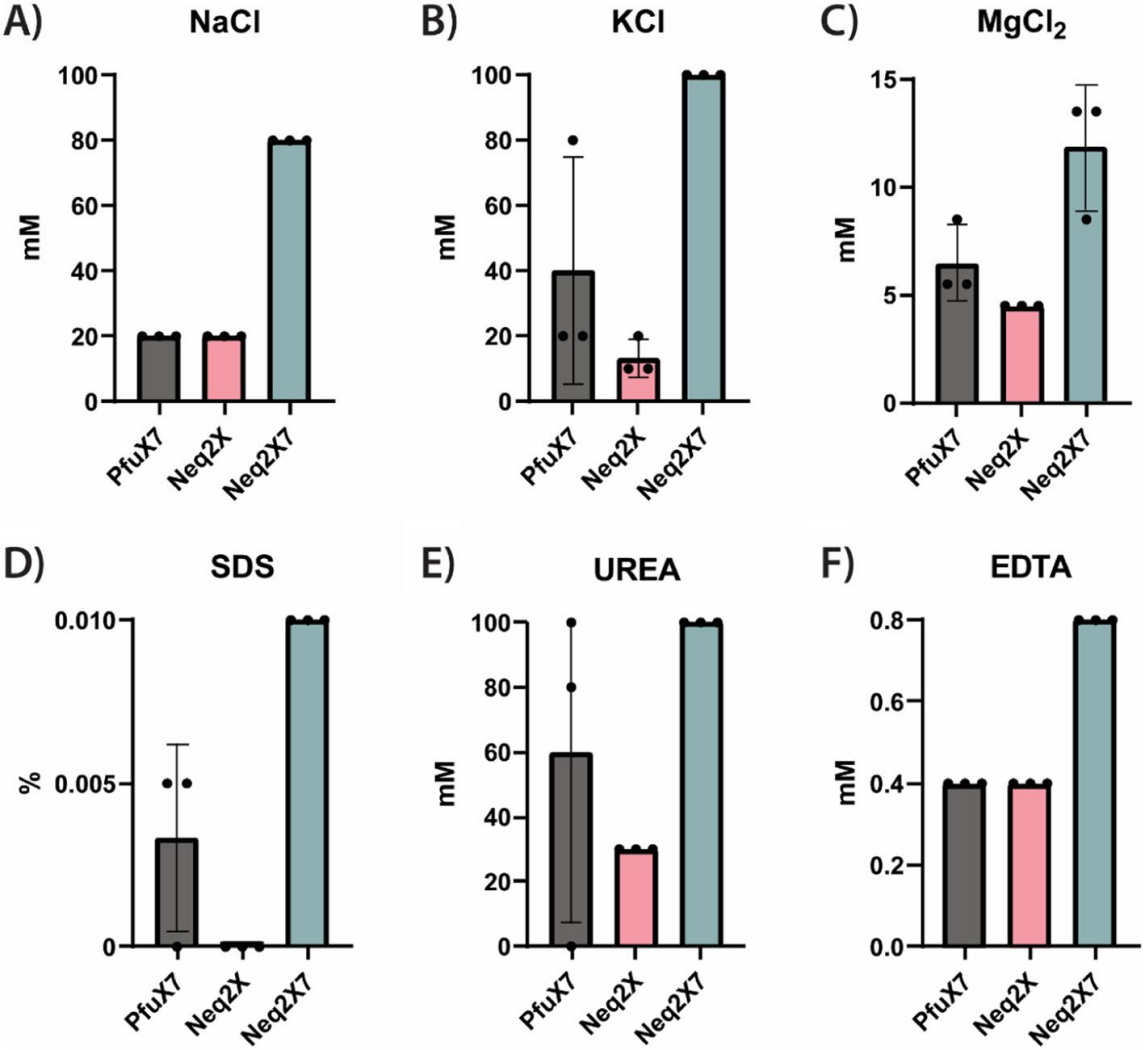
Inhibitory effect on the amplification of PfuX7, Neq2X, and Neq2X7 DNA polymerases by different substance concentrations. Maximum PCR amplification tolerance observed in the presence of (**A**) NaCl (mM), (**B**) KCl (mM), (**C**) MgCl_2_ (mM), (**D**) SDS (%), (**E**) Urea (mM), and (**F**) EDTA (mM) from three independent PCR reactions

PCR inhibitors can occur in environmental samples or can be introduced during sample preparation such as nucleic acid extraction and purification [39] and can significantly reduce DNA amplification. Such inhibitors interfere with PCR through various mechanisms, including disrupting primer binding, destabilizing DNA duplexes, impeding polymerase activity, and sequestering of metal ions [40]. Common inhibitory substances include sodium chloride (NaCl), magnesium chloride (MgCl_2_), potassium chloride (KCl), ethylenediaminetetraacetic acid (EDTA), sodium dodecyl sulfate (SDS), and urea. We investigated the effect of these chemicals on PfuX7, Neq2X, and Neq2X7 at inhibitory concentrations previously reported [40–42]. Three independent PCRs were conducted for each polymerase with different inhibitors using the same target DNA and primers (see materials and methods), and the average efficiency was determined by agarose gel electrophoresis. The results are presented in Fig. 4, along with Supplementary Figs. 7, 8, and 9. In the conditions tested in this study, Neq2X7 shows superior performance in overcoming the inhibitory effects of the substances investigated, especially when compared to Neq2X. Specifically, Neq2X7 displays a remarkable ability to tolerate 3–5 times more NaCl, KCl and MgCl_2_. Interestingly, we observed that the presence of SDS heavily impacts Neq2X, whereas Neq2X7 can amplify DNA in the presence of up to 0.01% SDS, indicating a distinct advantage conferred by the addition of Sso7d in tolerating SDS inhibition. Moreover, Neq2X7 tolerates urea up to 100 mM, while Neq2X reaches its inhibitory threshold at 30 mM. Notably, Neq2X and Neq2X7 activity is enhanced with the addition of EDTA – with 0.4 mM found to be the optimal for Neq2X and 0.8 mM for Neq2X7 (see Supplementary Figs. 8 and 9).

These results overall indicate that Neq2X7 is a very robust DNA polymerase able to amplify DNA across a range of conditions and even in the presence of PCR inhibitors. It should be noted that different types of templates and PCR applications will likely require individual optimization of the buffer conditions, and developing a custom buffer formulation for Neq2X7 could enhance its performance and fidelity for specific applications.

## Conclusions

The addition of the Sso7d binding domain to the double mutated (A523R/N540R) Neq DNA polymerase yields a more active DNA polymerase, Neq2X7, that in the last couple of years has proven to be a useful alternative to other available DNA polymerases in our laboratory. Here, we demonstrate Neq2X7 performance with long amplicons (12,000 bp), short extension time PCRs, and with dUTP replacing dTTP entirely in the dNTP mix commonly used in PCR. In a synthetic biology laboratory like ours, the latter is of particular interest in combination with the DNA assembly method known as USER cloning, but the enzyme could also find good use in forensic laboratories utilizing dUTP incorporation for prevention of template carry-over.

Neq2X7 has an improved ability to amplify high-GC DNA. However, we observed decreased fidelity of Neq2X7 specifically on G and C error positions. The higher fidelity of the Neq2X7 polymerase on A and T positions and the general difficulty of the Neq polymerase to handle high-GC content templates may relate to the high AT content of the *N. equitans* genome (31.6% GC) and highlights a potential weakness of this polymerase. The engineered Neq polymerase enables efficient amplification on high-GC templates, but probably a relatively high number of errors must be expected.

Even though there are several strategies to reduce and mitigate the inhibitory effect of different substances, they are still tedious, costly, and time-consuming. Therefore, a robust DNA polymerase able to tolerate a large range of PCR inhibitors is desirable. It is essential to acknowledge that specific tolerances to PCR inhibitors may vary based on factors such as DNA template origin, buffer composition, reaction conditions, and the presence of other components during the PCR reaction. The observation that Neq2X7 outperforms an enzyme like Pfu in some of the PCR applications tested here is an indication that the natural capabilities to accept uracil containing DNA is an attractive feature that should be further investigated in other thermostable organisms. When comparing Neq2X7 with the parental variant Neq2X, we observed a tradeoff between speed, inhibitor tolerance and fidelity for the two polymerases.

Our results highlight that Neq polymerase variants are useful additions to the toolboxes for diagnostic PCR and synthetic biology. Neq2X7 specifically is an efficient and versatile polymerase suitable for routine molecular biology as well specialty applications, like USER cloning, or prevention of PCR carry-over.

The Neq2X7 expression construct is available through the DNA repository Addgene, and the enzyme is easy and cheap to produce and purify using standard protocols. We hope that the sharing will facilitate its use in creating useful synthetic biology designs and biotech applications or in relevant teaching environments with limited resources.

## Methods

### Vector constructions

We used the pET-PfuX7 vector expressing PfuX7 DNA polymerase as described in [7], Addgene ID 182364. A pET vector expressing Neq2X DNA polymerase [25] with the Sso7d DNA binding domain fused to the polymerase C-terminus (See Fig. 1A) was synthesized by GenScript using the native DNA sequence, as used in [22] with the exception that the 6x-His tag was placed at the N-terminus, and we named it Neq2X7 (Addgene ID 182366). The sequence of the Neq2X7 fusion gene can be found in Supplementary Note S1. The Neq2X DNA polymerase vector (Addgene ID 182365) was created by removing the Sso7d DNA binding domain by USER cloning with primers: Forward_USER and Reverse_USER as described in [6] and cloned into *E. coli* NEB5α (New England Biolabs, Ipswich, MA, USA) competent cells. Oligonucleotides used in this study are listed in Supplementary Table 1. Strains used in this study are listed in Supplementary Table 2.

### Protein production

All three vectors were transformed into chemically competent *E. coli* Rosetta2 (DE3) pLysS (Novagen, Merck, KGaA, Darmstadt, Germany). As a note, attempts to generate PfuX7, Neq2X, and Neq2X7 using *E. coli* BL21 (DE3) were unsuccessful. Consequently, we suggest employing *E. coli* Rosetta2 (DE3) or similar strains for successful production of Neq2X7 following our established protocol. A single colony derived from the transformation was used to set an overnight culture in a 3 mL 2xYT medium (2x Yeast Extract Tryptone medium, Sigma Aldrich, Y2377) at 37 °C with 250 rpm of shaking. Cultures were supplemented with 100 µg/mL ampicillin and 25 µg/mL chloramphenicol for PfuX7 plasmid and 50 µg/mL kanamycin and 25 µg/mL chloramphenicol for Neq2X and Neq2X7 plasmids. The next day, the overnight culture was diluted 1:100 into 100 mL 2xYT media with the same antibiotics and growth conditions. When the culture reached an OD600 of 0.3, the expression was induced with 0.5 mM of IPTG. After four hours, the cultures were harvested, and the cell pellets were frozen at -80 °C until purification.

To lyse the cells, the pellets were slowly thawed on ice and resuspended in 4 mL of a lysis buffer containing 50 mM NaH_2_PO_4_, 300 mM NaCl, 10 mM imidazole, pH 8.0. 300 units per 100 mL of culture of Benzonase (250 U/µl), lysozyme (10 mg/mL), and EDTA free protease inhibitor cocktail were further added to the lysis buffer. The resuspension was kept on ice for two hours, followed by a heating step at 80 °C for 15 min. The heated mixture was centrifuged at 8000 g, at 4 °C for 20 min, and the supernatant containing the soluble fraction was collected and filtered sterilized before purification in the ÄKTA Pure chromatograph (GE Healthcare).

### Protein purification

PfuX7, Neq2X, and Neq2X7 were purified by affinity chromatography using a 1 ml HisTrap™HP on an ÄKTA Pure chromatography system (GE Healthcare). After washing of the column with 20 column volumes of wash buffer, the target protein was eluted using a gradient protocol and the following buffers: wash buffer: 50 mM NaH_2_PO_4_, 300 mM NaCl, 20 mM imidazole (pH 8.0) and elution buffer: 50 mM NaH_2_PO_4_, 300 mM NaCl, 500 mM imidazole (pH 8.0). A linear gradient was applied, increasing the percentage of elution buffer from 0 to 100% over 18 column volumes at a flow rate of 1 mL/min. Peak fractions were analyzed on an InstantBlue (abcam) stained SDS-PAGE gel (4–20% Mini-PROTEAN^R^ TGX™, Bio-Rad). Fractions containing the polymerase were pooled and concentrated using an Amicon® Ultra Centrifugal Filter, 10 kDa MWCO, followed by a desalting step on a PD-10 desalting column (Merck), before loading them on a preparative Superdex200 increase 10/300 GL (GE Healthcare) column for size exclusion chromatography using a running buffer containing 20 mM Tris-HCl, 10 mM KCl, 6 mM (NH_4_)2SO_4_, 2 mM MgSO_4_ (pH 8.8). Peak fractions were analyzed on an InstantBlue (abcam) stained SDS-PAGE gel and subsequently pooled (see Supplementary Fig. 12). Samples were initially diluted 1:2 in storage buffer (20 mM Tris-HCl (pH 8.8), 10 mM KCl, 6 mM (NH_4_)2SO_4_, 2 mM MgSO_4_, 0.1 mg/mL BSA (nuclease free), 0.1% Triton X-100 and 50% glycerol) and subsequently stored at -20 °C.

Protein concentration was determined by densitometry using the Fiji software [43] as the BSA-containing buffer in the purified polymerase sample does not give accurate protein concentration measurement, and standard protein quantification methods could not be used. Triplicate representative sample preparations were loaded on an SDS-PAGE gel for each polymerase (PfuX7, Neq2X, and Neq2X7) containing 5 µL of the sample + 5 µl of sample buffer. We used a serial dilution of a protein with a known protein concentration to estimate the protein concentration, which we used to calculate the standard curve, from which we figured the protein concentration of PfuX7, Neq2X, and Neq2X7 (see Supplementary Fig. 2A, C and D).

We wish to emphasize that a simple, standard benchtop affinity purification protocol is sufficient to produce large amounts of Neq2X7 suitable for routine PCR applications. Additionally, a basic protein dilution is enough to determine the optimal working concentration for the benchtop purified polymerase.

### DNA polymerase activity assay

To determine the polymerase activities of PfuX7, Neq2X, and Neq2X7, we followed the fluorescence method described by [44] with minor modifications. In a 15 µL solution containing 1.2 pmol of the single-stranded M13mp18 DNA (Bionordika) previously annealed with 1.6 pmol of the primer UPlong (IDT) was mixed with 0.2 mM mixture of each dNTP (dATP, dCTP, dGTP, and dTTP), 2.5 µL of 5xHF buffer (Thermofisher Scientific) and water to 15 µL. We used three different DNA polymerase protein concentrations (0.5, 1, and 3 pmol) to determine the DNA polymerase activity, adjusted to a final volume of 5 µL. After ssDNA-primer-dNTPs-buffer temperature equilibration at 72 °C, we added 5 µL containing the polymerase, and the reaction was placed back at 72 °C in a PCR cycler for 1, 3, and 15 min. Triplicate replicates were used of each concentration and time point for statistical analysis. The reaction was terminated by adding 1 µL of 0.5 mM EDTA, following the addition of 103.8 µL TE buffer (10 mM Tris-HCl, pH 7.9, 50 mM KCl, 1 mM EDTA) and 0.2125 µL Pico488 dsDNA quantification reagent (Lumiprobe) to a final volume of 200 µL per sample. The 200 µL mixture was transferred to a 96-well black plate, and after 4-minute incubation, the absorbance was measured at 485 nm excitation and 528 nm emission with a top gain of 70. Double-stranded DNA of a known concentration measured by a Qubit 2.0 fluorometer (Invitrogen) were used to fit a linear regression to calculate the DNA amplification produced by the polymerases (Fig. 1D, E, F).

### PCR conditions

All PCR conditions unless stated otherwise were run using the following standard PCR protocol: DNA denaturation step of 98 °C for 3 min, and 30 cycles of a second denaturation step at 98 °C for 30 s, a primer annealing step of 60 °C for 30 s, and an amplification step at 72 °C with variable times. Oligonucleotide sequences and templates are listed in Supplementary Tables 1, 3, 4, and 5. To test the effect of different PCR inhibitors, the following conditions were used to amplify a target sequence of 641 bp on a pET plasmid using primers Fw_1 and Rv_2): an initial denaturation step at 98 °C for 4 min, followed by thirty cycles of a 98 °C denaturation step of 10 s, annealing at 55 °C for 30 s and elongation at 72 °C for 15 s, with a final extension at 72 °C for 10 min),

### Amplification of GC-rich DNA

Three templates Scat1, Scat2, and Tth2 of high GC content (see Supplementary Table 4), were chosen to test DNA polymerase performance. A touchdown PCR consisting of the following steps was used: an initial denaturation step of 98 °C for four minutes, followed by 10 cycles of 98 °C for 45 s, annealing at 65 °C with one degree decrement every cycle, and an extension time of 1 min, afterward followed by 30 cycles of 98 °C for 45 s, annealing at 55 °C, and extension of 1 min. Primer pairs, templates and GC content are listed in Supplementary Table 4.

### Polymerase fidelity assay

We followed the protocol for the MagNIFI assay as presented by de Paz et al. with some modifications [36]. We used the same template sequences as designed by de Paz et al. The extension primer and duplex adapter sequences were modified to be compatible with the binding sites of the Unique Dual Index Kit (Takara Bio, #63752) for Illumina TruSeq library generation (Supplementary Table 1). Oligonucleotides were ordered from Integrated DNA Technologies (IDT). Each template was annealed with the extension primer by mixing 70 nM primer with 105 nM template (1:1.5 molar ratio) in 1x HF buffer (NEB, B0518S). The mixture was incubated at 95 °C for 2 min, followed by a ramp of -1 °C/10 sec until reaching 4 °C.

For the extension reactions, we prepared all reaction conditions in duplicates, using the same annealed template for each pair of biological replicates. We tested six concentrations of the rare dNTP in the range from 10 µM to 10 − 4 µM. 500 µM stock solutions (50x) containing three out of four dNTPs were prepared from individual 100 mM stocks (Thermo Fisher). Extension reactions for Neq2X7, Neq2X, and Phusion polymerase (Thermo Fisher, F549S) were set up using 5x HF buffer (NEB, B0518S). Extension reactions using Taq polymerase (Thermo Fisher, EP0401) were set up using Taq buffer with KCl and supplemented with 1.5 mM MgCl_2_. Primer extension reactions were prepared in a final volume of 25 µl containing 2.5 µl of the annealing reaction, 1x DNA polymerase reaction buffer, 10 µM of three non-rare dNTPs, and variable concentrations of the rare dNTP and variable DNA polymerase units (Phusion: 0.5 U, Taq: 0.625 U, 0.5 U Neq2X7, 0.5 U Neq2X). The reactions were incubated at 72 °C for 1 h.

### Illumina library preparation and sequencing

The individual primer extension reactions were purified using the ZR-96 DNA Clean & Concentrator-5 kit (96-well format, Zymo Research, D4024). The purified DNA fragments were eluted in 18 µl of nuclease-free water. Then, a 22 bp duplex adapter, modified with a 5’ phosphate modification, containing the sequence for the universal binding site for the Unique dual indexing TruSeq kit (Takara Bio) was blunt-end ligated to the 3’ end of the purified extension reaction products. 6 µl of purified extension product were mixed with 1 µl of 10x T4 ligase buffer (Thermo Fisher), 1.5 nM adapter, and 2.5 Weiss units T4 ligase (Thermo Fisher, EL0011) in a 10 µl reaction. The ligation reaction was incubated at 22 °C for 1 h and subsequently heat inactivated at 65 °C for 10 min.

Samples were barcoded and PCR amplified in a 20 µl reaction using 1x Phusion High-Fidelity PCR Master Mix with HF Buffer (Thermo Fisher, F531), 2 µl of ligation product, and 400 nM of forward and reverse primers from the Unique Dual Index kit (1–96) (Takara Bio, #63752). Each pair of unique index primers was used for two samples, pairing either A and C templates or G and T templates. This pairing was demultiplexed during the alignment to the different template sequences. The PCR program was as follows: Initial denaturation for 30 s at 98 °C, 20 cycles of 10 s at 98 °C, 30 s at 60 °C and 30 s at 72 °C followed by a final extension at 72 °C for 10 min. A subset of samples was analyzed on 1% agarose gels to verify successful amplification and correct fragment size.

The barcoded PCR products were purified using MagBind Total Pure NGS beads (Omega BioTek, M1378) in an automated set-up using a Bravo liquid handling platform (Agilent) and eluted in 25 µl 10 mM Tris-HCl buffer (pH 8). The concentrations of all individual samples were measured in a microplate reader using the Pico488 dsDNA Quantification Reagent (Lumiprobe) as described above. Based on these measurements, all samples were normalized to a concentration of 10 ng/µl in a 15 µl volume. The samples were then pooled into four separate pools (one per polymerase) and the sample quality and fragment size was assessed using a 2100 Bioanalyzer (Agilent) using the High Sensitivity DNA assay (Supplementary Fig. 6B). The concentration of each library pool was verified using a Qubit 2.0 Fluorometer (Life Technologies) before pooling all samples into one combined library pool and diluting to a final concentration of 10 nM based on an estimated average fragment size of 300 bp. Samples were stored at -20 °C in-between processing steps.

The library pool was mixed with 35 % phiX, diluted and denatured according to MiSeq System Denature and Dilute Libraries Guide from Illumina. The sample was then sequenced using MiSeqV2 Nano 300 cycles kit. The raw reads with bcl files were processed and demultiplexed using basespace (Illumina) according to Unique Dual Indexes used for each sample. We obtained on average 3011 ^+^/- 568 reads per barcode (Supplementary Fig. 10).

### Error rate analysis

Forward and paired-end reverse reads were filtered for the presence of the 22/30 bp template sequences flanking the error enrichment site (Supplementary Table 6) and trimmed down to the 59 bases containing these flanking regions, the variable context, and the error enrichment site. The filtering and trimming was performed using cutadapt v4.4 [45] in paired-read mode for linked, non-anchored adapter sequences (-g/-G) with the following options: action = retain noindels -O = 10. Since the template sequences for the T and C templates are very similar, the maximum error rate for a match to the flanking sequences was fixed at e = 1 for these templates. The filtered read pairs were then tested for a complete match between the forward read and the reverse complement of the paired read. The required 100 % match between forward and reverse read excludes potential sequencing errors at the error enrichment site since the identical error would have to occur in both paired reads to pass the filter. Final quality assessment and filtering was performed using fastp v0.22 [46] to remove reads with more than 10% of low quality (Q < 15) bases and pose additional requirements for the correct final read length (50–70) (A unqualified_percent_limit = 10 --length_required = 50 --length_limit = 70). The sequencing quality at the error enrichment site was consistently Q > 30. After a read pair passed the filters, the following analysis steps were only performed on the forward read. Reads were aligned to the full set of all four template sequences using bowtie2 v2.2.5 in local alignment mode [47]. Statistics about matches, mismatches and single nucleotide deletions at the error enrichment site were obtained using the samtools v1.6 mpileup tool [48, 49]. The fit of the dose-response curve was performed in GraphPad Prism v10.0 with the following settings: variable slope, four parameters, least squares (ordinary) fit, constraining the bottom and top value to 0 and 1, respectively. Error rates [bp^−1^] were calculated using the following equation: ER = 10^(2.063 ∙^ ^*log*^^(FC50) +1.557)^ [36].

### Supplementary Information

**Supplementary Material 1.**

## Data Availability

An earlier version of the manuscript is available at bioRxiv at https://www.biorxiv.org/content/10.1101/2022.03.14.484273v1. Plasmids and plasmid sequences are available at Addgene.
