# Supplementary material for: Neq2X7: a multi-purpose and open-source fusion DNA polymerase for advanced DNA engineering and diagnostics PCR
Source: BMC Biotechnol. 2024 Apr 2;24:17. doi: 10.1186/s12896-024-00844-7 (PMC10988834; doi:10.1186/s12896-024-00844-7)
Supplement: Supplementary file 1 — Supplementary Material 1. [file 12896_2024_844_MOESM1_ESM.docx]

**Supplementary information for:**

Neq2X7: a multi-purpose and open-source fusion DNA polymerase for advanced DNA engineering and diagnostics PCR

Cristina Hernández-Rollán^1,#^, Anja K. Ehrmann^1,#^, Arsenios Vlassis^1^, Vijayalakshmi Kandasamy^1^, and Morten H. H. Nørholm^1, 2^*.

^1^The Novo Nordisk Foundation Center for Biosustainability, Technical University of Denmark, Søltofts Plads Building 220, 2800 Kongens Lyngby, Denmark

^2^Mycropt ApS, 2800 Kongens Lyngby, Denmark

^#^ CHR and AKE contributed equally

*Corresponding author

Addresses of all authors

Cristina Hernández Rollán: cristina.hernandez@cpr.ku.dk

Anja K. Ehrmann: anjaeh@biosustain.dtu.dk

Arsenios Vlassis: arsvla@biosustain.dtu.dk

Vijayalakshmi Kandasamy: vijkan@biosustain.dtu.dk

Morten H. H. Nørholm: morno@biosustain.dtu.dk

# Supplementary Figure 1

**Supplementary Figure 1.** Number of citations in Google scholar for the keywords Taq, Pfu, and Neq DNA polymerases represented on a log scale.

#
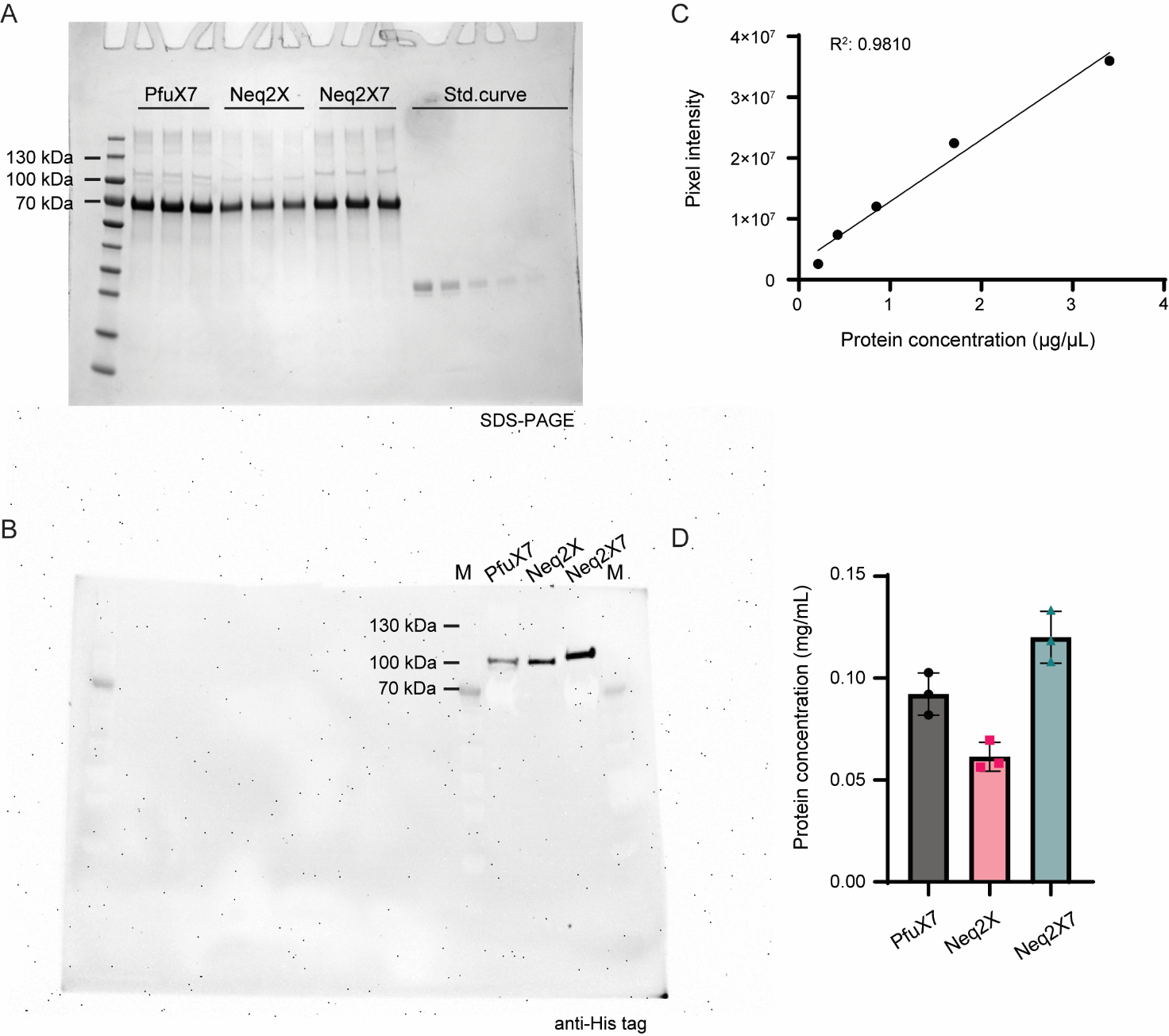
Supplementary Figure 2

**Supplementary Figure 2.** **Estimation of the purity and concentration of the PfuX7, Neq2X, and Neq2X7 polymerases following Ni-NTA affinity and gel filtration purification**. (A) Coomassie blue-stained SDS–PAGE gel after Ni-NTA purification of PfuX7, Neq2X, and Neq2X7 polymerases in triplicates alongside protein standards utilized for estimating protein concentrations via Fiji software^28^. (B) Western blot using a His-tag antibody verifying the presence of the three DNA polymerases (C) The protein standards with known concentrations were used to establish a linear fit, enabling the estimation of PfuX7, Neq2X, and Neq2X7 protein concentrations. (D) The PfuX7, Neq2X, and Neq2X7 protein concentrations in mg/mL were calculated based on the linear regression fit. The molecular weights of the polymerases are 97.6 kDa for PfuX7, 95.7 kDa for Neq2X, and 103.2 kDa for Neq2X7.

# Supplementary Figure 3


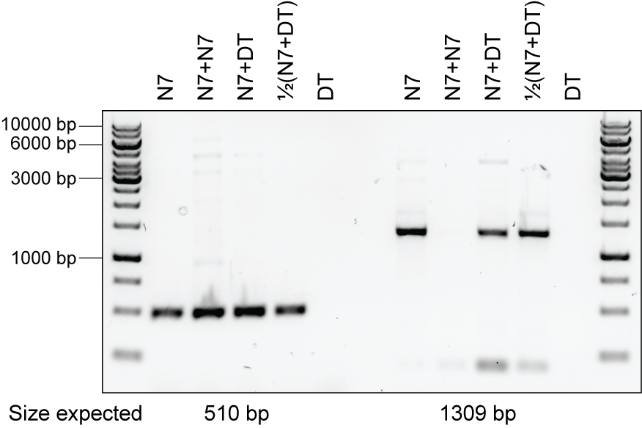


**Supplementary Figure 3.** **Evaluation of PCR performance of a mixture of Neq2X7 and DreamTaq DNA polymerases**. Two regions of the *E. coli* genome were amplified using either 1 pmol Neq2X7 (N7), 2 pmol Neq2X7 (N7 + N7), 1 pmol Neq2X7 + 0.625 U DreamTaq (Thermo Scientific) (N7 + DT), 0.5 pmol Neq2X7 + 0.31 U DreamTaq (½(N7+DT)) or 0.625 U DreamTaq (DT) in 25 µl reactions. Primers used: melB_fw, melB_rv (510 bp amplicon), recA_fw, recA_rv (1309 bp amplicon).

## **Supplementary Figure 4**

## **
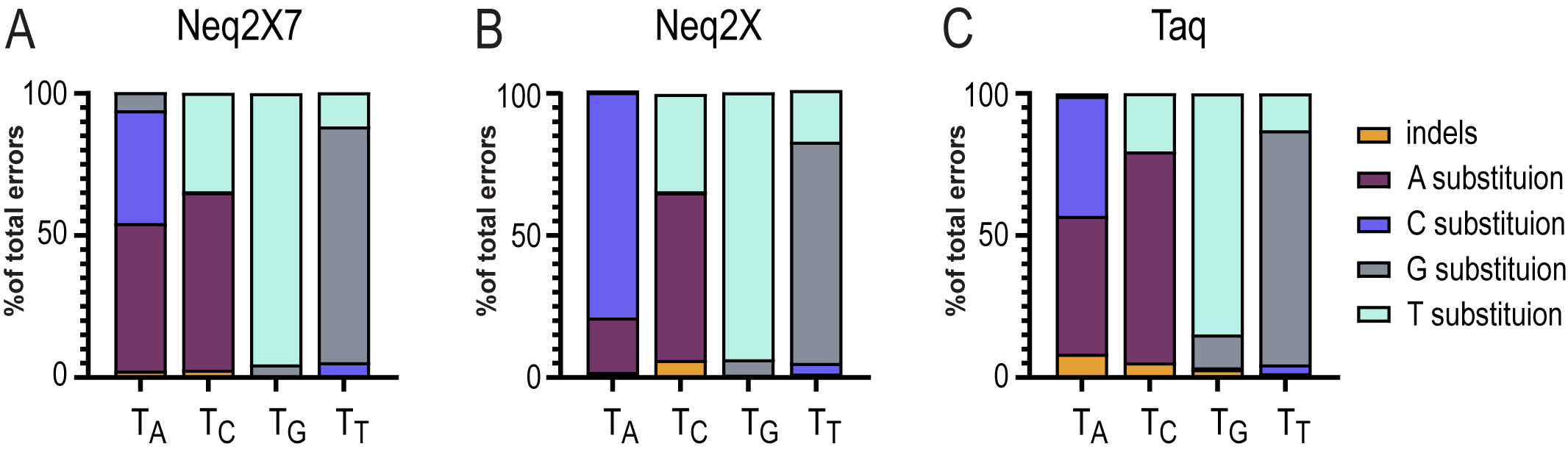
**

**Supplementary Figure 4.** Detailed error profiles for A) Neq2X7, B) Neq2X and C) Taq. Frequency of different base substitutions and single nucleotide deletions relative to the total number of errors at the error enrichment site resolved by the four different template contexts for each polymerase at 10^‑4^ µM rare dNTPs. The error profile for Phusion polymerase was not analyzed due to the overall low number of observed errors.

## **Supplementary Figure 5**

## **
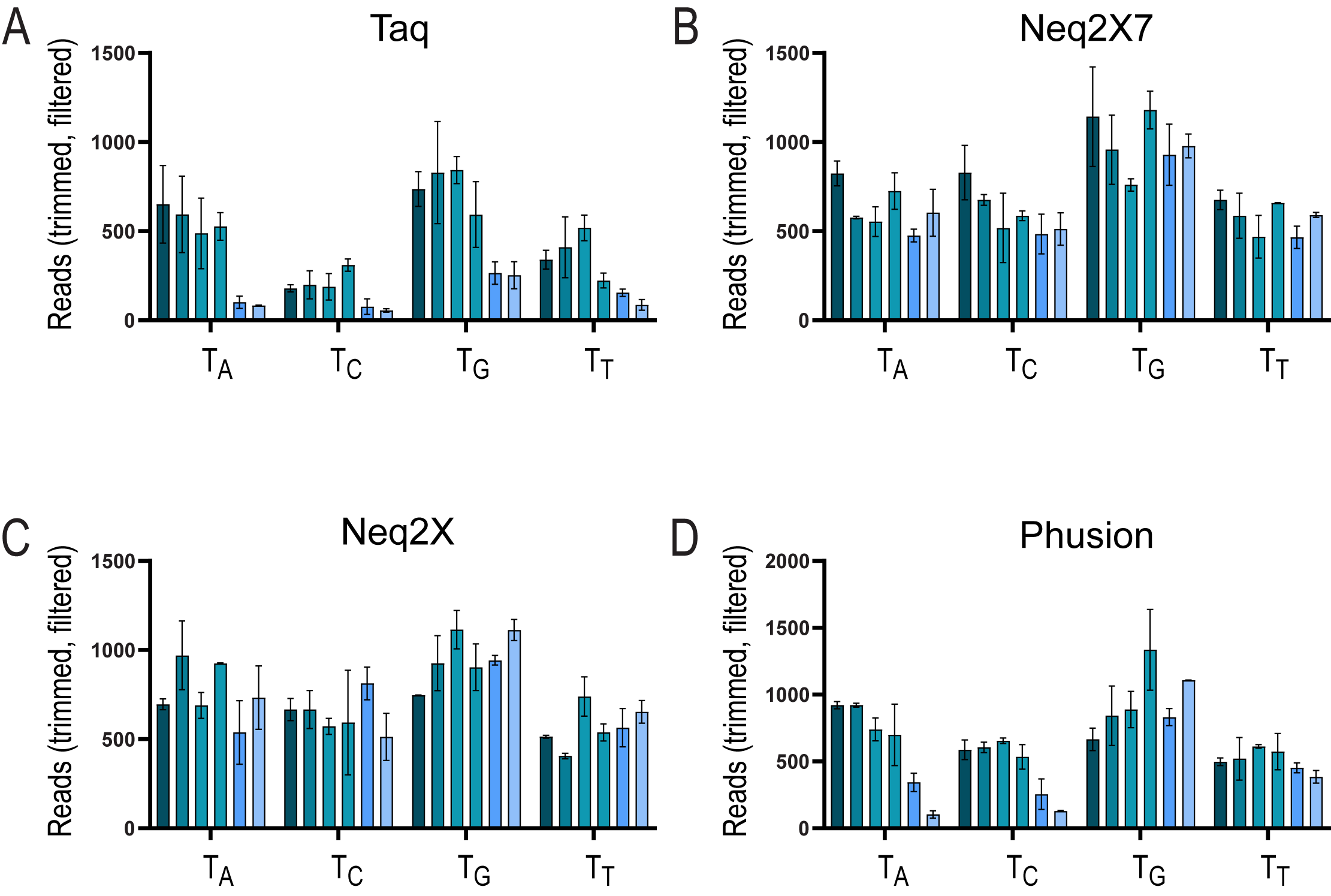
**

**Supplementary Figure 5.** Number of reads obtained after trimming and filtering for each sample condition. Bars represent the mean of two replicates, error bars the standard deviation.

## **Supplementary Figure 6**

## **
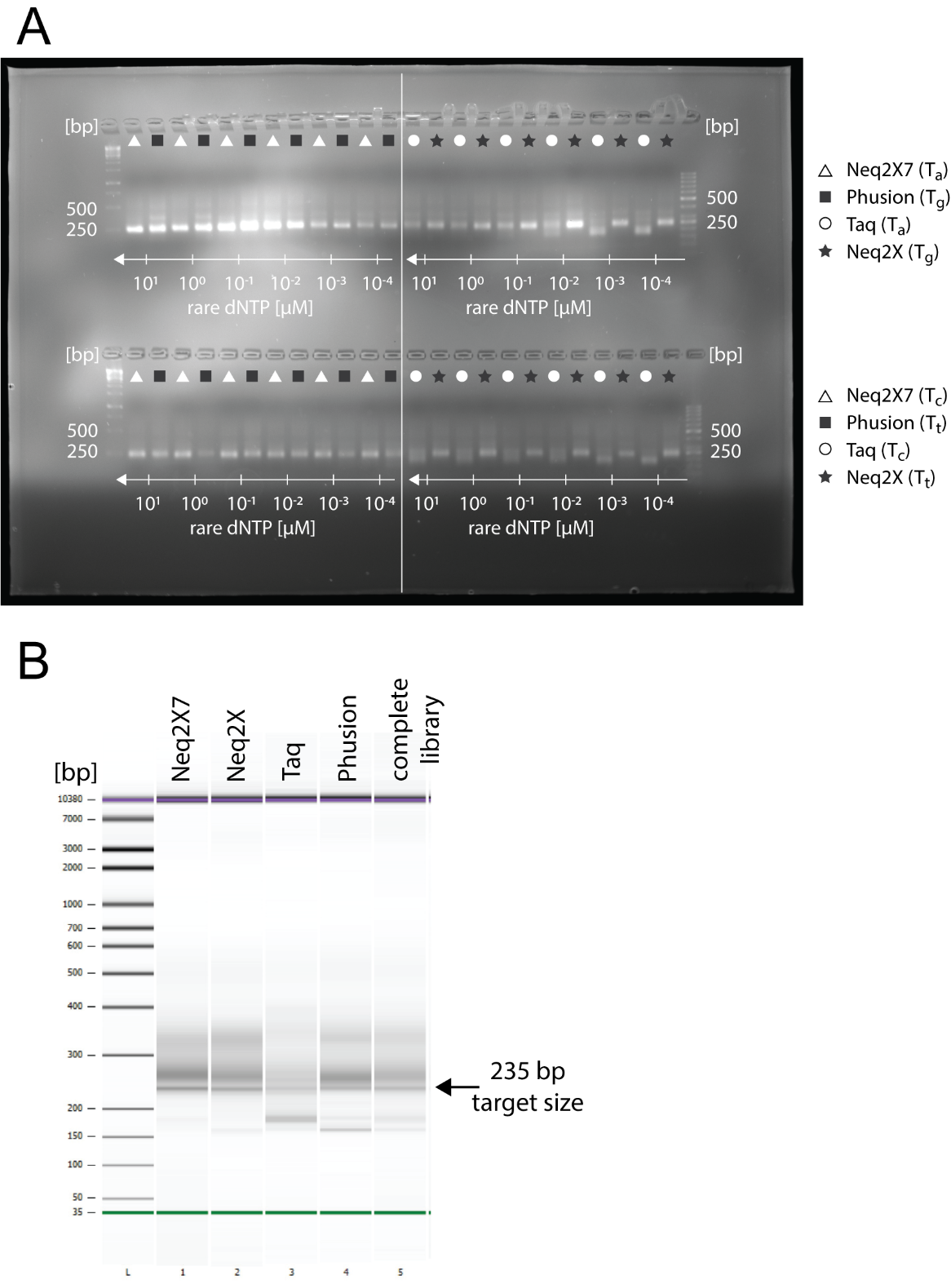
**

**Supplementary Figure 6.** A) Gel image showing the fragment size of selected amplicons after library preparation for sequencing**.** The expected fragment size is 235 bp. B) Size distribution of DNA amplicon libraries for sequencing (MagNIFI assay)

**Supplementary Figure 7**
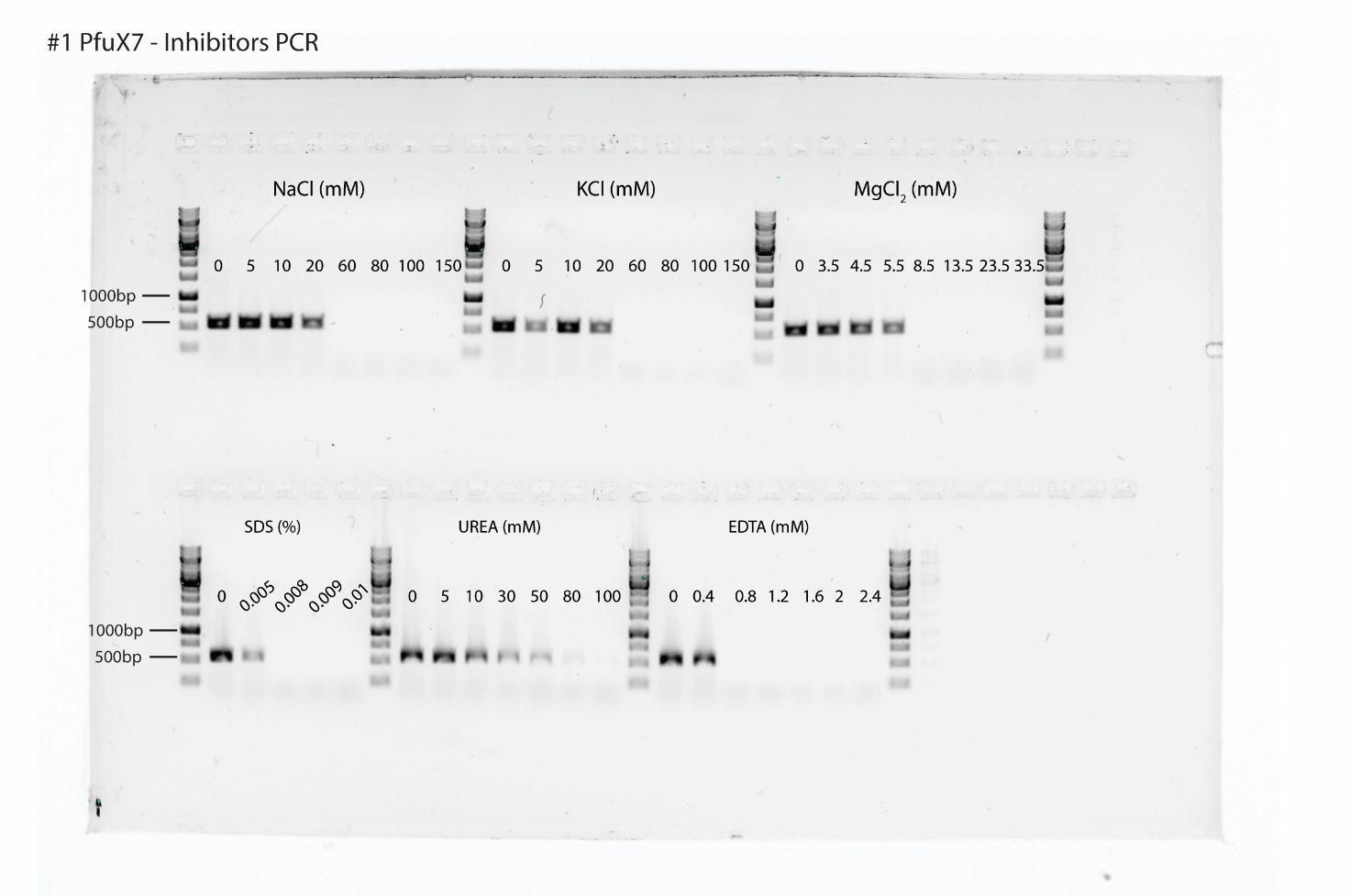

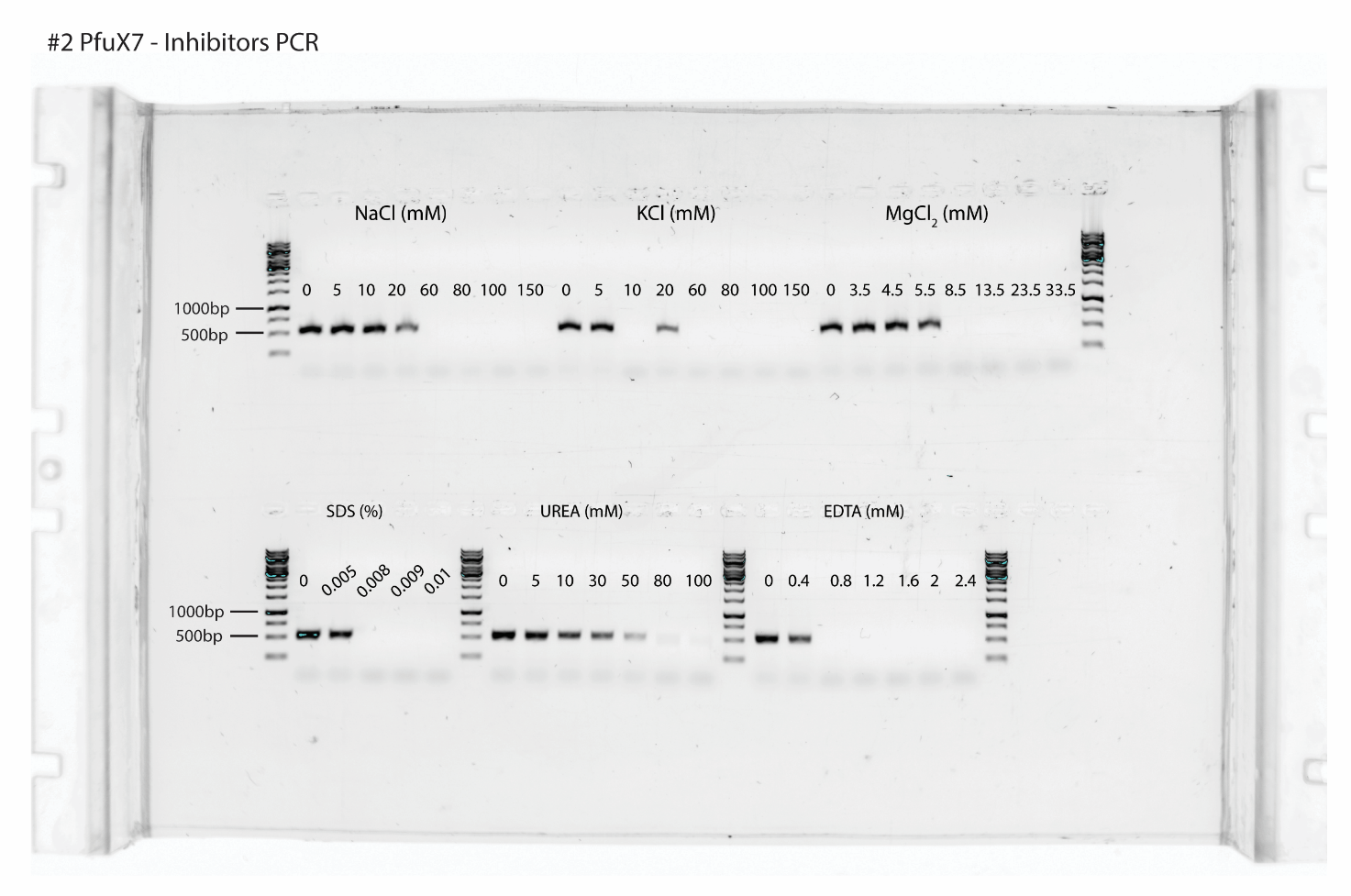


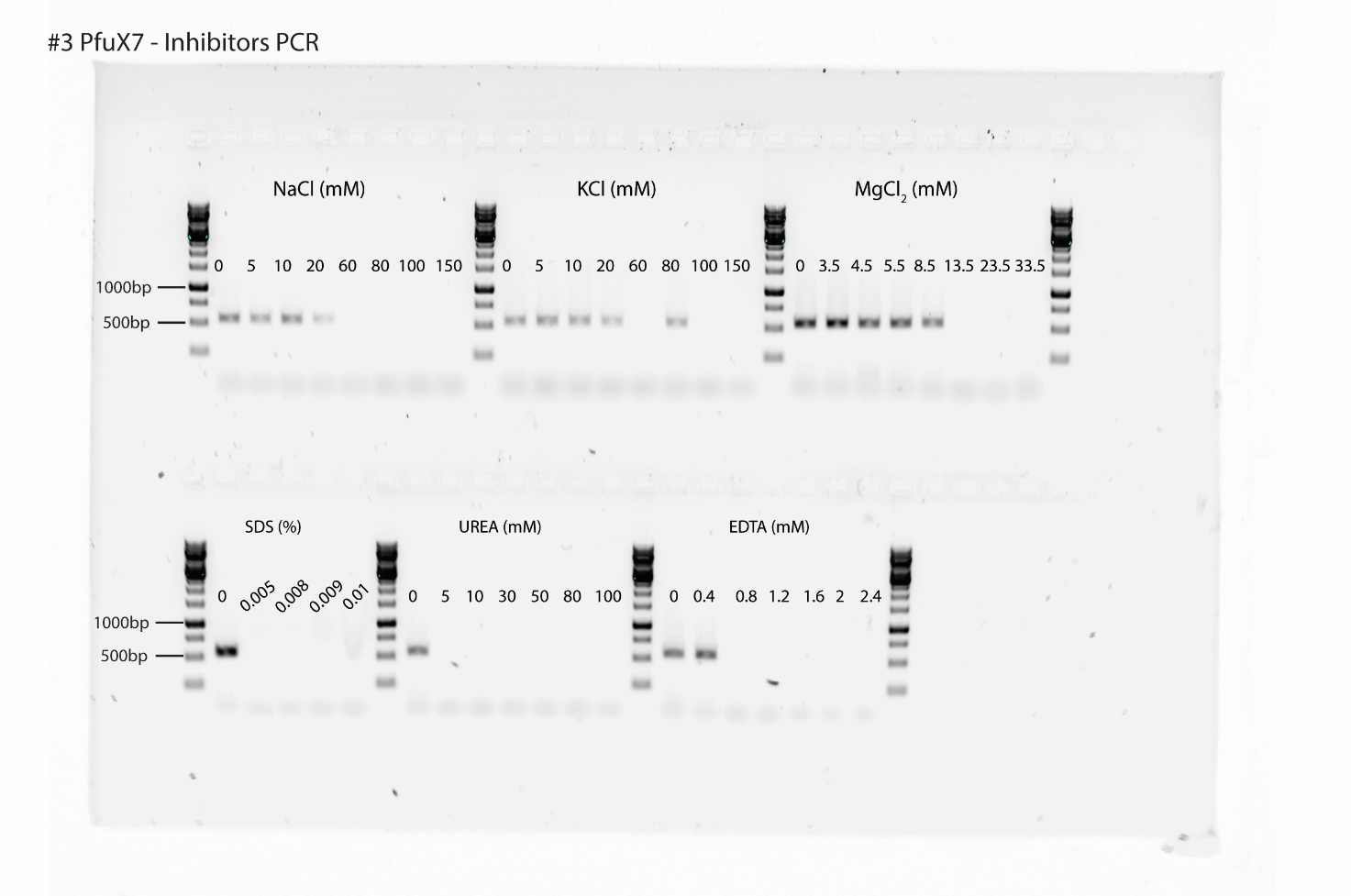


**Supplementary Figure 7. Evaluation of the inhibitory effect of common PCR inhibitors on PCR amplification by PfuX7 DNA polymerase.** Agarose gel electrophoresis of the amplification of a target DNA template (641 bp) utilizing increasing amounts of PCR inhibitory substances by the PfuX7 DNA polymerase.

**Supplementary Figure 8**


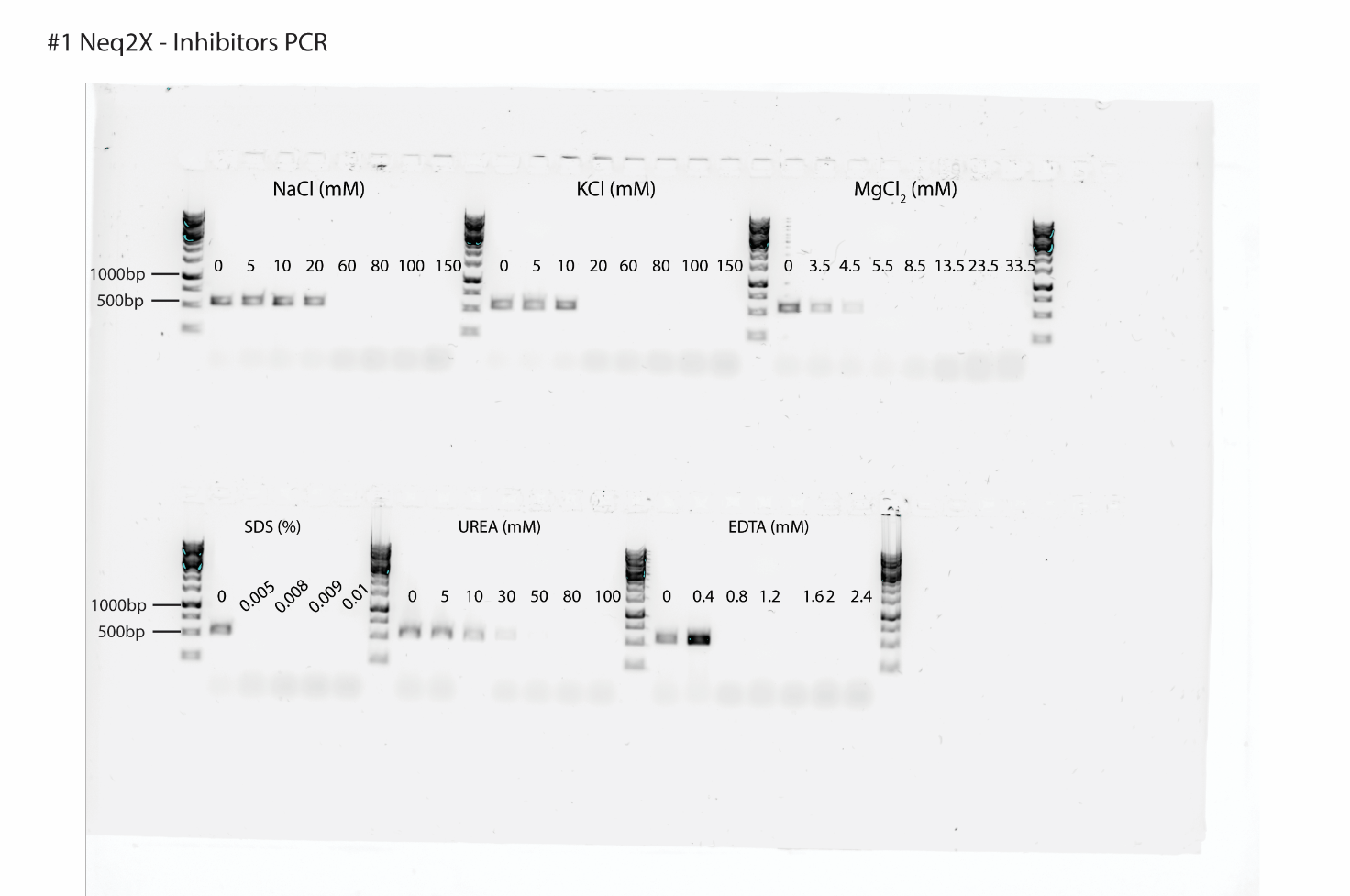

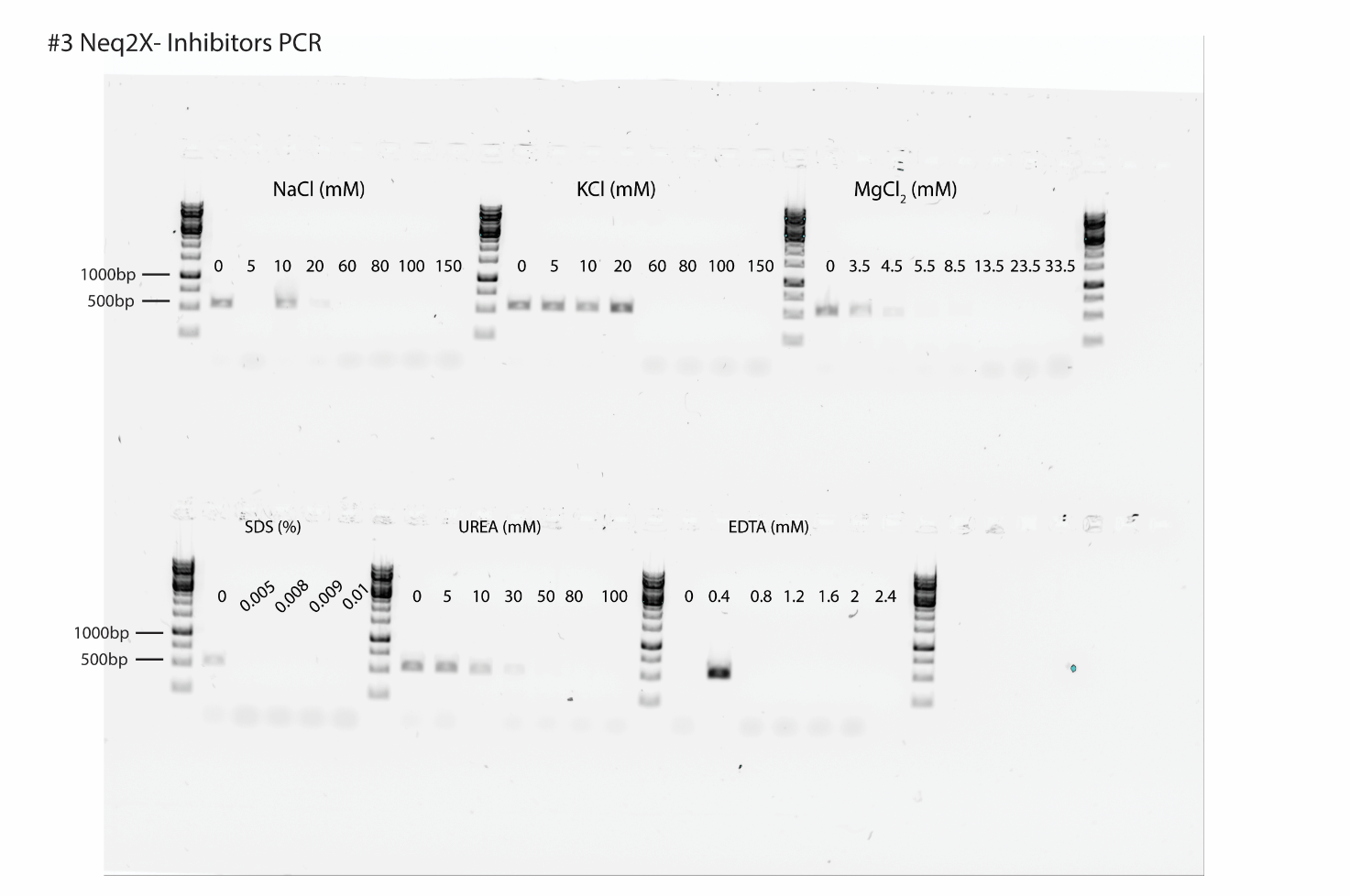


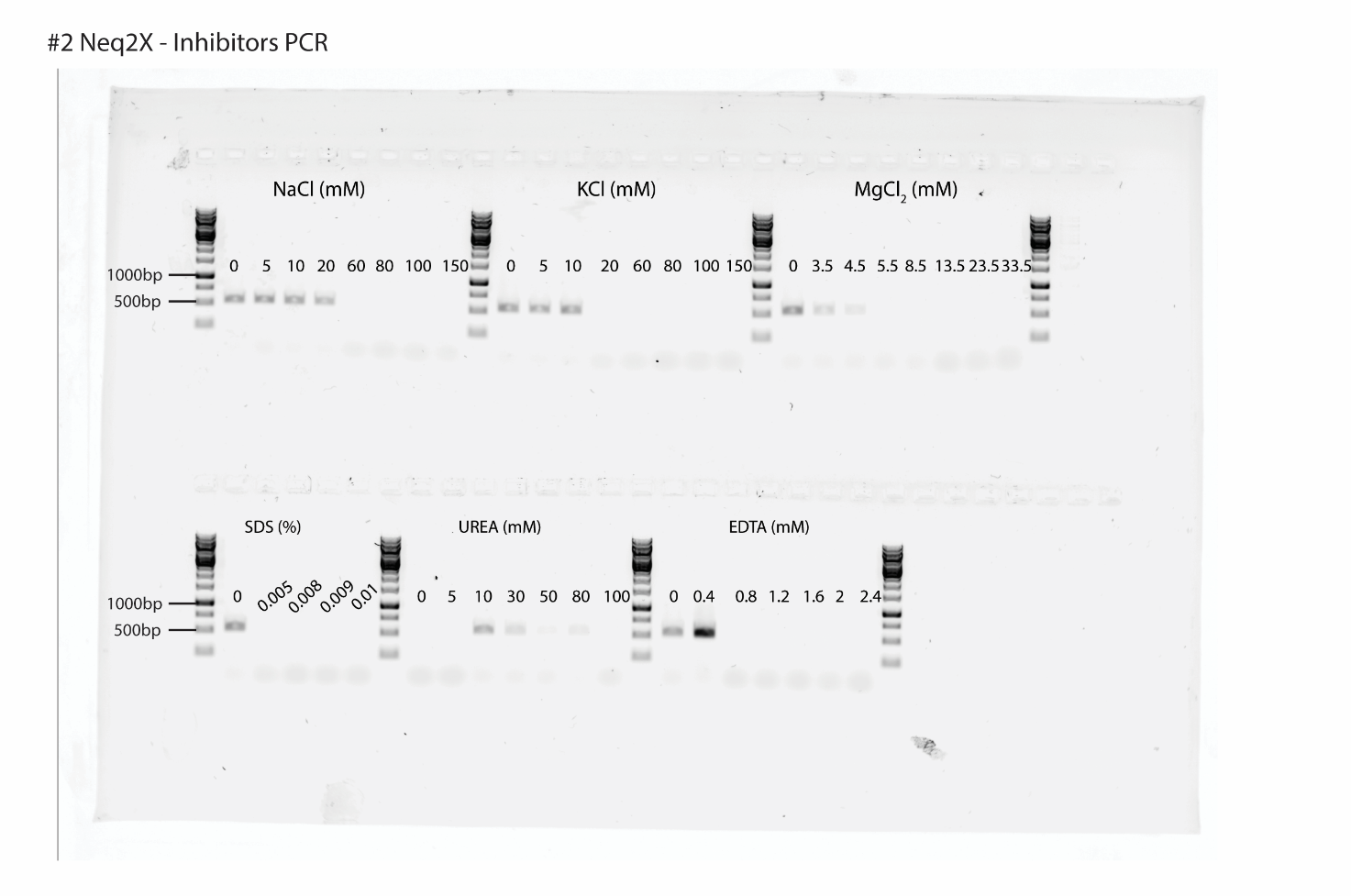


**Supplementary Figure 8. Evaluation of the inhibitory effect of common PCR inhibitors on PCR amplification by Neq2X DNA polymerase.** Agarose gel electrophoresis of the amplification of a target DNA template (641 bp) utilizing increasing amounts of PCR inhibitory substances by the Neq2X DNA polymerase.

**Supplementary Figure 9**


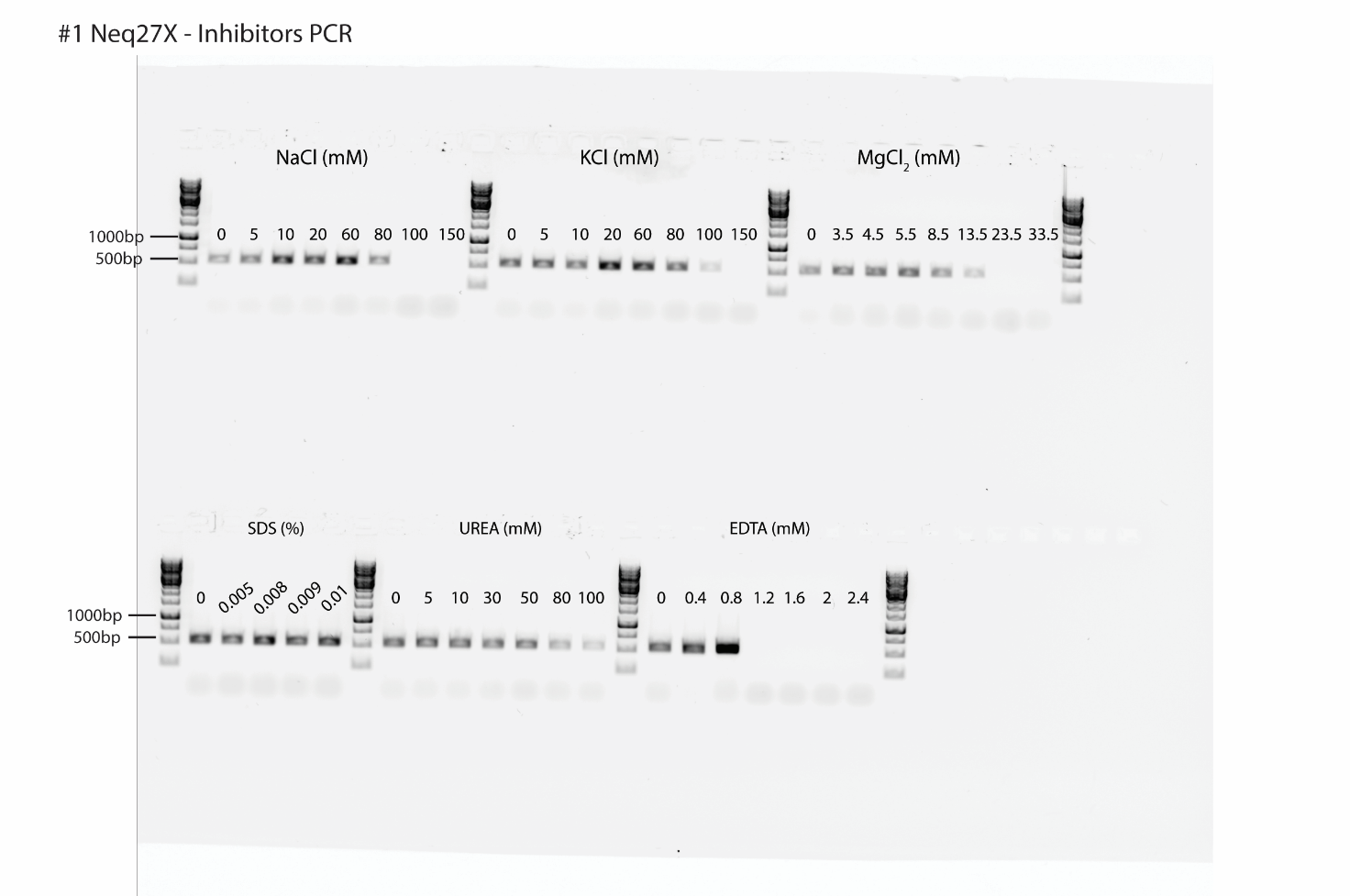

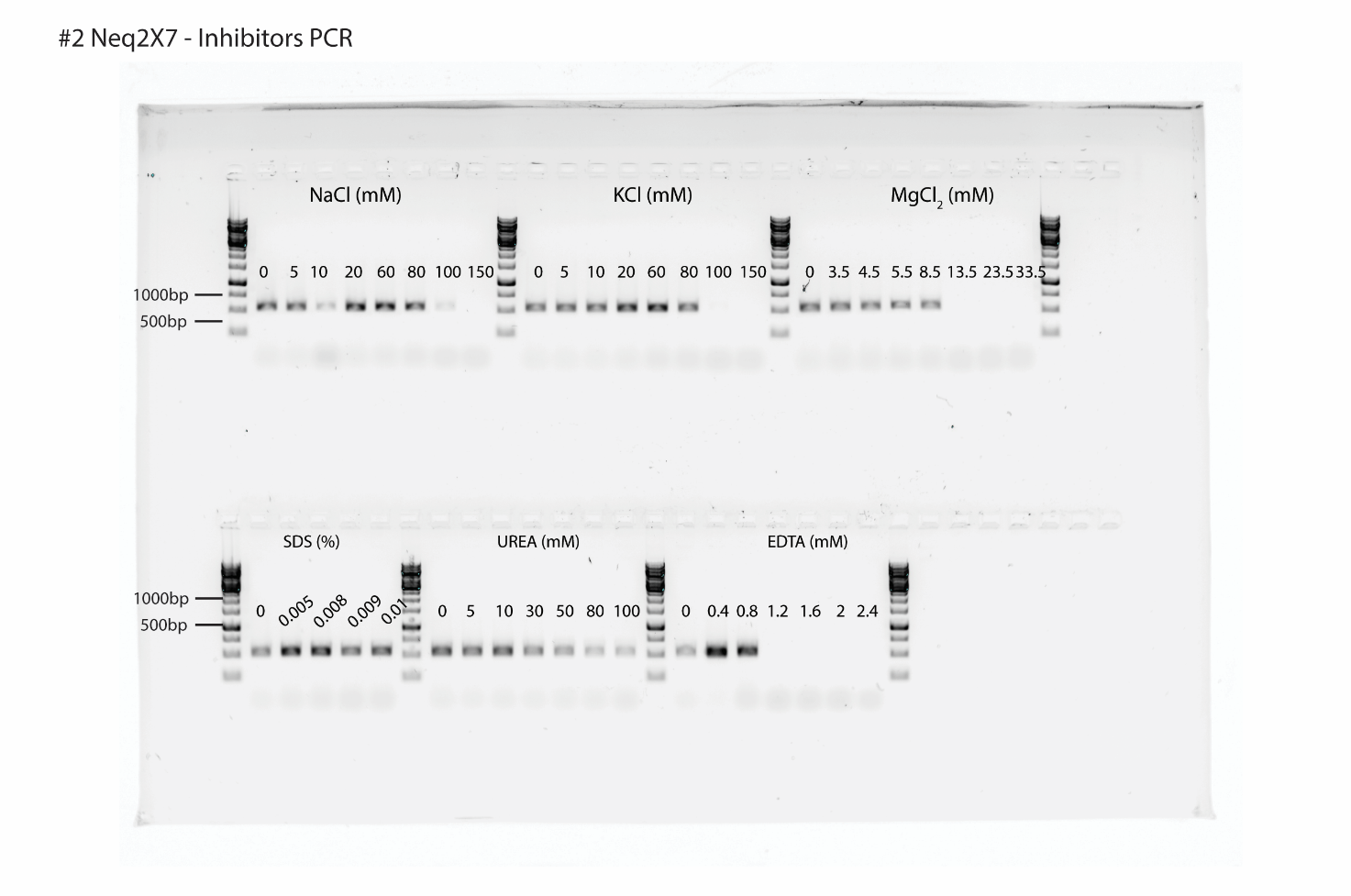


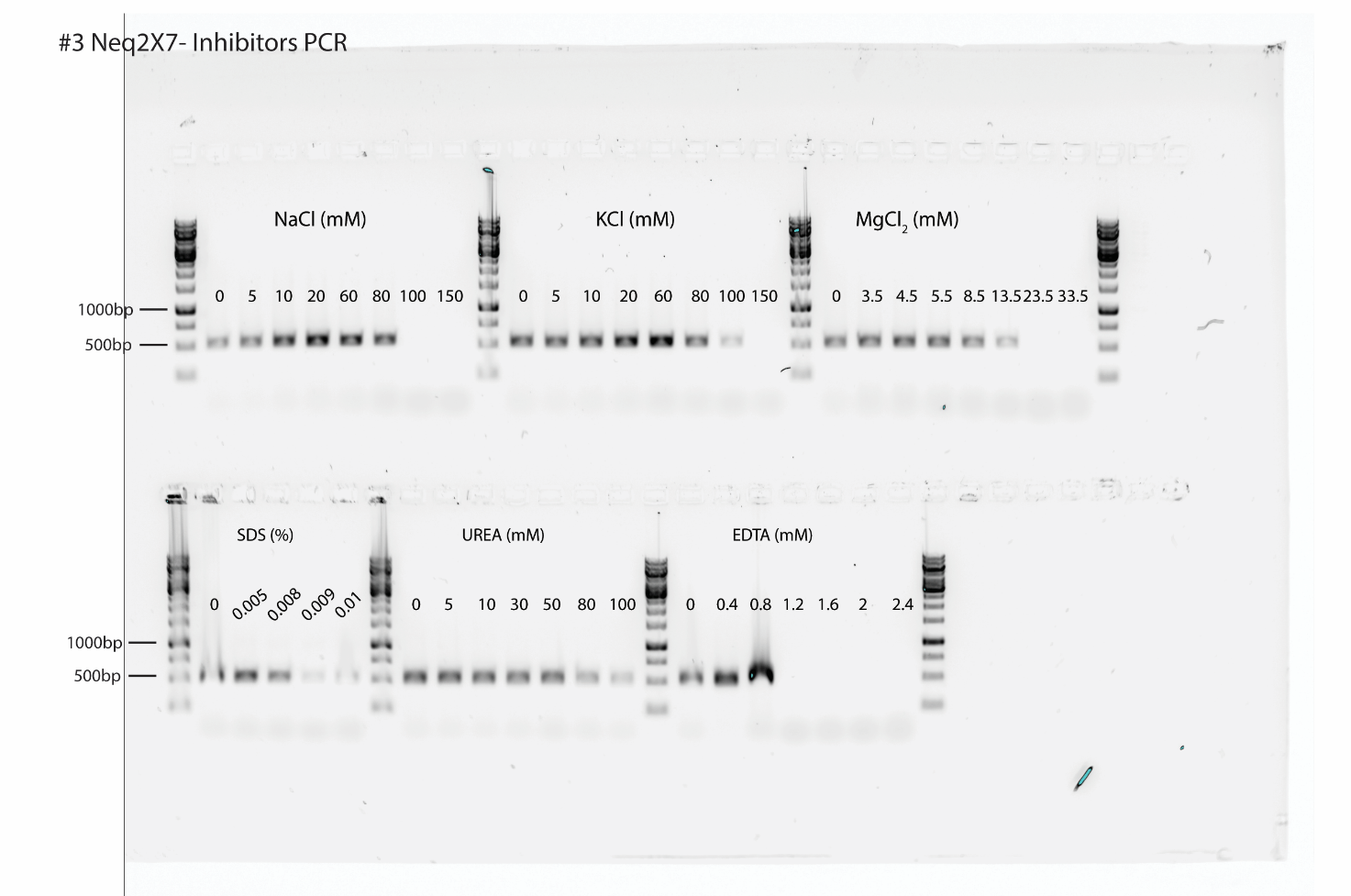


**Supplementary Figure 9. Evaluation of the inhibitory effect of common PCR inhibitors on PCR amplification by Neq2X7 DNA polymerase.** Agarose gel electrophoresis of the amplification of a target DNA template (641 bp) utilizing increasing amounts of PCR inhibitory substances by the Neq2X7 DNA polymerase.

**
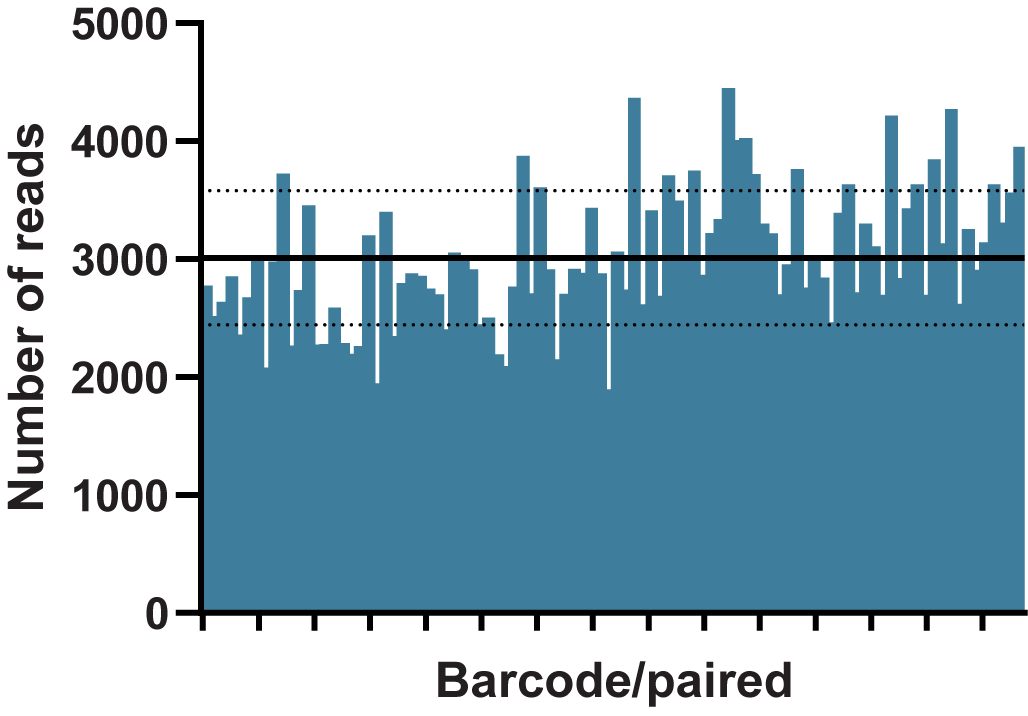
Supplementary Figure 10**

**Supplementary Figure 10.** Reads passing filter per barcode after Illumina sequencing for the MagNIFI assay.

**Supplementary Figure 11**

**
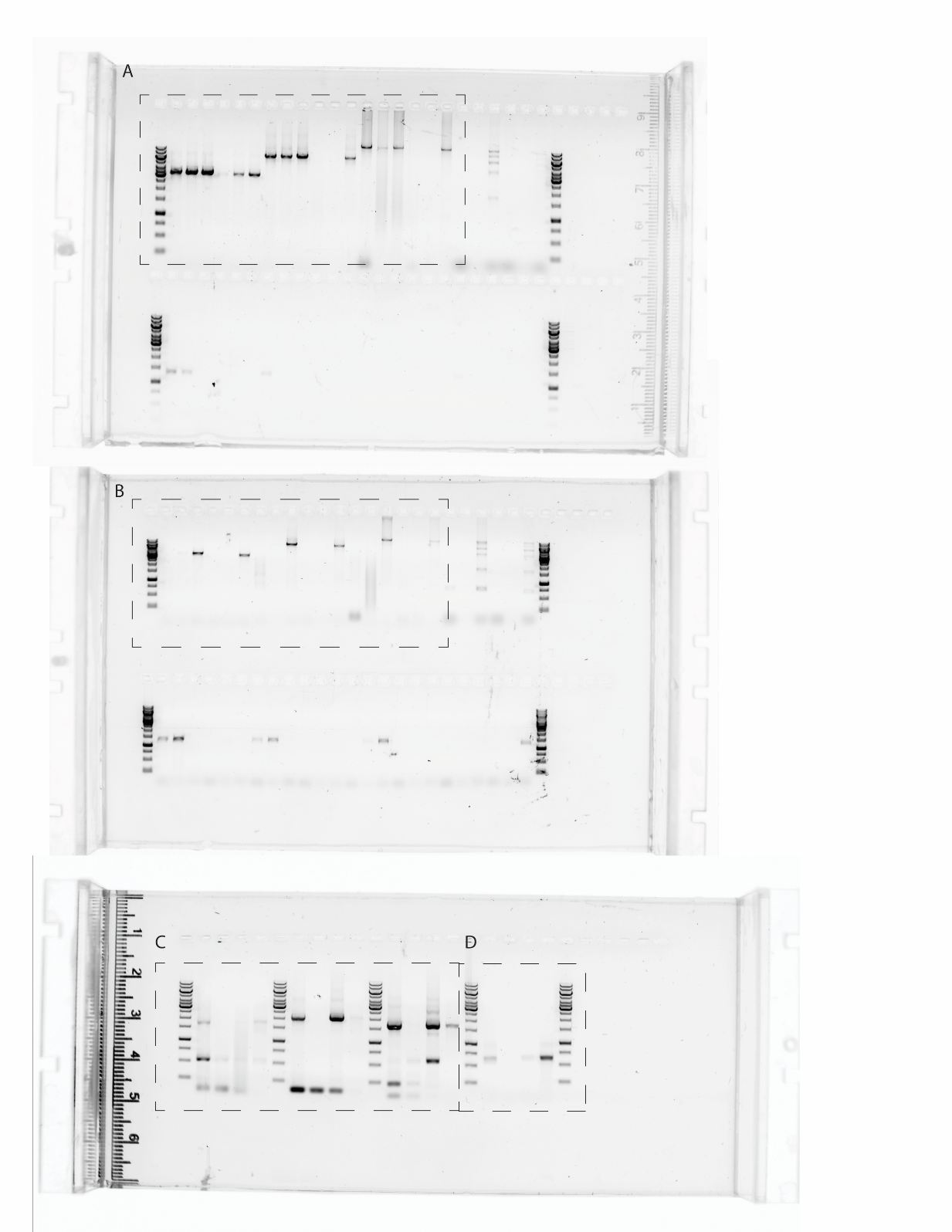
**

**Supplementary Figure 11.** Uncropped gels from Figure 2. The boxes represent the portion cropped used in the main figure for clarification purposes.

**Supplementary Figure 12**

**
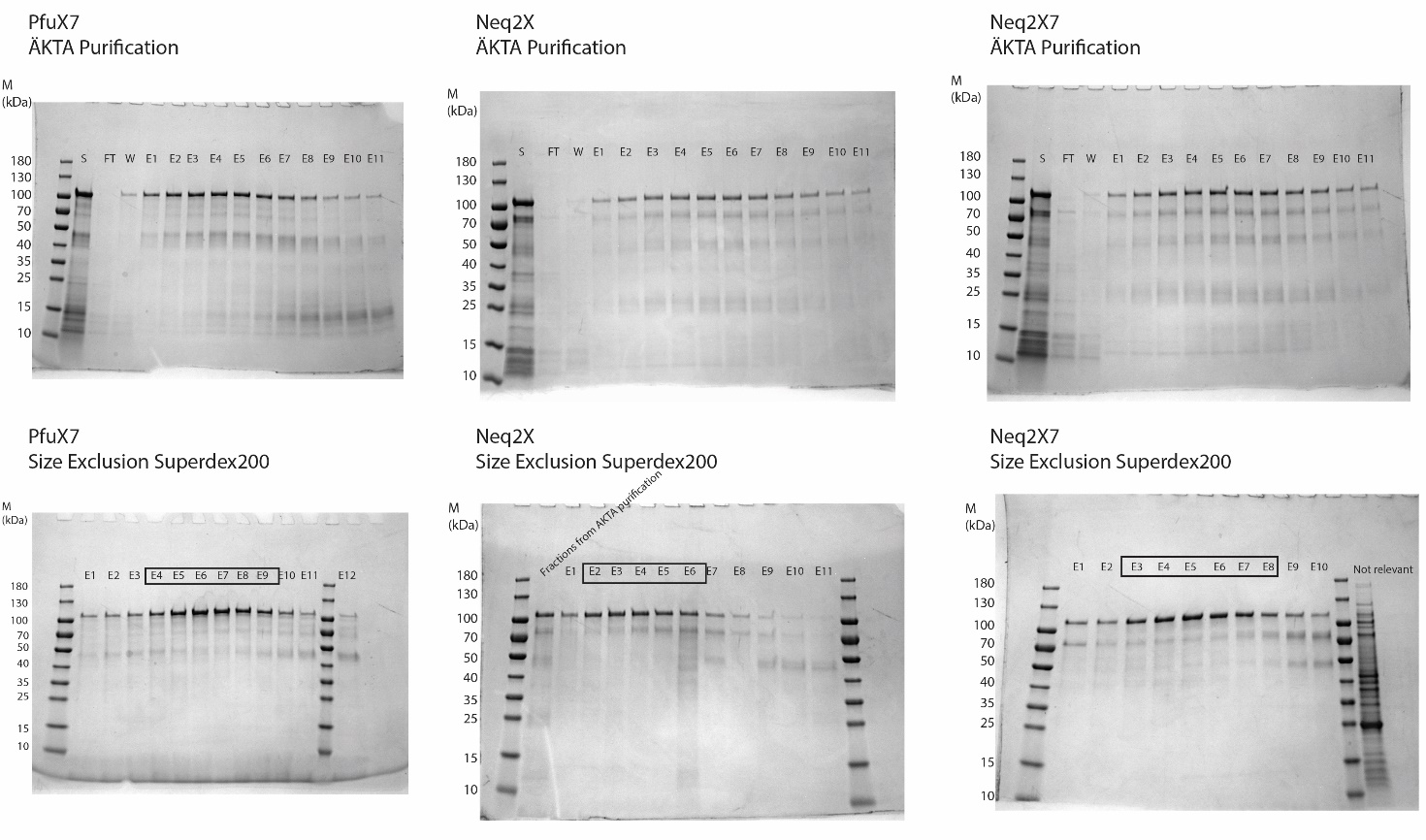
**

**Supplementary Figure 12.** Coomassie blue-stained SDS–PAGE gels showing the ÄKTA purification and size exclusion chromatography for PfuX7, Neq2X, and Neq2X7. Final pooled fractions for PfuX7, Neq2X, and Neq2X7 are highlighted with a square. M (kDa) Marker in kDa. S (sample before purification), FT (flow-through fraction), W (wash fraction), E (elution fractions).

# Supplementary Table 1

**Supplementary Table 1. Oligonucleotides used in this study.**

| **Name** | **Sequence (5' --> 3')** |
| --- | --- |
| Fw_1 | TAATACGACTCACTATAGGGGAATTG |
| Rw_2 | GCTCAGCGGTGGCAGCAGCCAACTCAGCTT |
| Fw_3 | AAACGCTGTCTTGGAACCTA |
| Rv_4 | GCAAATGGCATTCTGACATCC |
| Rv_5 | ACGGCTTCAGAATTTCTCAAGAC |
| Rv_6 | AAACTGTCAGTTTTGGGCCAT |
| Fw_7 | CGTATGCATTGCAGACCTTGTGG |
| Rv_8 | GCACGATCCAACAGGCGAGC |
| Fw_9 | TATGGTGGTGAAGGGCGGTTC |
| Forward_USER | AAATAAGAGAUCCGGCTGCTAACAAAGC |
| Reverse_USER | ATCTCTTATTUAAAGAAATCTGTTAGTTTTTTGCCTGTGAATGTG |
| Fw_Scat1_73% | AGGCGAUAGGAGGAATATACATGAGTCCTACGCCGCACA |
| Rv_Scat1_73% | AGCCGUGCAGCACGGTCAGCGTGGT |
| Fw_Scat2_76.6% | ACGGCUCCAACCTGGGCGCCTGCCGGGA |
| Rv_Scat2_76.6% | GGTGCGAUTCACCCGGCGGCGTACACGT |
| Fw_Tth2_66.3% | AGAACCUCGAGGGCGACGAGCTGAAGA |
| Rv_Tth2_66.3% | GGTGCGAUTTAGTCAAAGACATCTGTGGCATACC |
| melB_fw | TATGGTGGTGAAGGGCGGTTC |
| melB_rv | CGACGGTGATATTCCTCGCTC |
| recA_fw | CGTATGCATTGCAGACCTTGTGG |
| recA_rv | GCACGATCCAACAGGCGAGC |
| P_Ext_ | CTACACGACGCTCTTCCGATCTGGCTAGTCGTCTGTATAGG |
| Ligation adapter | /5Phos/AGATCGGAAGAGCACACGTCTG |
| TruSeq_Read1_UDI_P5 | AATGATACGGCGACCACCGAGATCTACACNNNNNNNNACACTCTTTCCCTACACGACGCTCTTCCGATCT |
| TruSeq_Read2_UDI_P7 | CAAGCAGAAGACGGCATACGAGATNNNNNNNNGTGACTGGAGTTCAGACGTGTGCTCTTCCGATCT |
| **Templates MagNIFI Fidelity Assay** | |
| T_T_ | GGAGAACACCCAAAACAACACCAAACAGCAAACAAAAAGGAGAGAGAAGAAVVVTVVVAAGGAAAGGAAAGAAGCGGAGACCTATACAGACGACTAGCC/3ddC/ |
| T_A_ | GGTGTTCTCCCTTTTCTTCTCCTTTCTCCTTTCTTTTTCCTGTGTGTTGTTBBBABBBTTGGTTTGGTTTGTTGCGGTGTCCTATACAGACGACTAGCC/3ddC/ |
| T_G_ | CAACAACTCCTAACTCAACAACTAACATCCAACCTTTCTCATATCCACCAAHHHGHHHAACCAAACCAAACAACCCCACACCTATACAGACGACTAGCC/3ddC/ |
| T_C_ | GAAGAAGTGGTAAGTGAAGAAGTAAGATGGAAGGTGTGTGATATGGAGGAADDDCDDDAAGGAAAGGAAAGAAGGGAAGACCTATACAGACGACTAGCC/3ddC/ |

# Supplementary Table 2

**Supplementary Table 2. Strains used in this study.**

| Strain | Genotype | Source/Reference |
| --- | --- | --- |
| *E. coli* NEB5α | *fhuA2* Δ(*argF-lacZ*)U169 *phoA* *glnV44* Φ80 Δ(*lacZ*)M15 *gyrA96 recA1 relA1 endA1 thi-1 hsdR17* | *a* |
| *E. coli* Rosetta BL21(DE3) | F-*ompT* *hsdS*_B_(r_B_- m_B_-) *gal dcm* (DE3) pLysSRARE2 (Cam^R^) | *b* |

*^a^*NEB, Ipswich, MA, USA.

*^b^*Novagen, Merck KGaA, Darmstadt, Germany.

# Supplementary Table 3

**Supplementary Table 3. PCR-fragments.** Template was a pPIC9K derived plasmid.

| Fragment | FW primer | RV primer |
| --- | --- | --- |
| 3300 bp | Fw_3 | Rv_4 |
| 6500 bp | Fw_3 | Rv_5 |
| 12000 bp | Fw_3 | Rv_6 |

# Supplementary Table 4

**Supplementary Table 4. PCR products and primers used for GC amplification PCR.**

| Gene | FW primer | RV primer | GC content | PCR product |
| --- | --- | --- | --- | --- |
| Scat1 | Fw_Scat1_73% | Rv_Scat1_73% | 73% | 1540 bp |
| Scat2 | Fw_Scat2_76.6% | Rv_Scat2_76.6% | 76.6% | 1680 bp |
| Tth2 | Fw_Tth2_66.3% | Rv_Tth2_66.3% | 66.3% | 1516 bp |

# Supplementary Table 5

**Supplementary Table 5. Fast PCR protocol and primers used in this study.**

| Step | Temperature | Time (seconds) | Cycle |
| --- | --- | --- | --- |
| DNA denaturation | 98°C | 40 | 1 |
| DNA denaturation | 98°C | 2 | 20 |
| Annealing | 60°C | 2 |  |
| Extension | 72°C | 20 |  |
| Primers | Fw_1 | Rv_2 |  |

# Supplementary Table 6

**Supplementary Table 6. Sequence context used to extract error enrichment site from raw reads**

| forward read | |
| --- | --- |
| T_t-fwd | TCTCCGCTTCTTTCCTTTCCTT;e=1...TTCTTCTCTCTCCTTTTTGTTTGCTGTTTG |
| T_a-fwd | ACACCGCAACAAACCAAACCAA...AACAACACACAGGAAAAAGAAAGGAGAAAG |
| T_g-fwd | TGTGGGGTTGTTTGGTTTGGTT...TTGGTGGATATGAGAAAGGTTGGATGTTAG |
| T_c-fwd | TCTTCCCTTCTTTCCTTTCCTT;e=1...TTCCTCCATATCACACACCTTCCATCTTAC |
| paired-end read | |
| T_t-rev | CAAACAGCAAACAAAAAGGAGAGAGAAGAA...AAGGAAAGGAAAGAAGCGGAGA |
| T_a-rev | CTTTCTCCTTTCTTTTTCCTGTGTGTTGTT...TTGGTTTGGTTTGTTGCGGTGT |
| T_g-rev | CTAACATCCAACCTTTCTCATATCCACCAA...AACCAAACCAAACAACCCCACA |
| T_c-rev | GTAAGATGGAAGGTGTGTGATATGGAGGAA...AAGGAAAGGAAAGAAGGGAAGA |

**Supplementary Note S1**

**Supplementary Note S1.** Sequence of the Neq2X7 gene including N-terminal 6xHis-tag and Sso7d DNA binding domain. Refer also to Addgene entry 182366.

atgcatcaccatcaccatcacggatcaatgttacaccaactccccacgatggttgtagaagaaaaggcggtaaaagaggaagaagggtatagcgtgctaaaatgttattggattaatatagagaacacccctttagacgaggtaattttaataggtaaagacgaaaataatagagcttgtgaagttataattccatacaaatggtatttctattttgaaggcgatataaaggatttagaagaattcgctaacaacaaaaaaataaaaatcgaatatacaaaggagcaaaagaaatatatagaaaaaccaaaagatgtttataaagtatatgttttgcataaacattatccaatactaaaagaattcattaaagaaaagggctataaaaaatacgaaaccgatataaatgtttataggaagtttttaatagataaagggatagagccttttgaatggtttgaggtagaaggcaaaattttattatctacctctaacaaagttagaataaaagcacaaagtataaaaagattgtatgaaaagactaagccatcggttttagcttttgatatagaagtttacagtgaggctttccctaatcctgaaaaagacaaaataatatctatagccctttatggagacaattacgaaggggttatctcttacaaaggagaaccaactataaaagttaataccgaatatgaattaattgagaaatttgtcgaaataatagaaagcttaaaaccagacataatagttacatacaatggggataatttcgatatagactttttagtgaaaagggcttctttatacaatataaggctaccaataaaattggttaacaaaaaagagcctacttataattttagggaaagcgcacatgtagatttgtataaaacaattactaccatatataaaacccaattgtctacccaaacatattcattaaatgaagtagctaaagaaattcttggagaggagaaaatttatgattatgaaaacatgttatatgattgggccataggcaattataacaaagtgttcgaatacaatttaaaagatgccgaattaacatataagctattcaaatactatgaaaatgatttattggaattagcaagattggttaaccaaccattatttgatgtatctaggtttagctatagtaatatagttgaatggtatctaatcaaaaaaagcagaaaatataatgaaattgtgcctaacaaaccaaaaatggaagaagtagagagaagaaaattaaatacctatgcaggagcattcgtttacgaaccaaaacccggtttgtatgagaatttagctgtactggatttcgcttctctgtatccttcaattatattagagcataatgtttctccaggcacaatatattgtgagcatgatgattgtaaacaaaatggggtagaagcgataataaataatgagaaaaaatatgtgtggttttgcaaaaaagtaaaagggtttattccaacggtattagagcatttgtatacaaaaaggctagaacttaagagaaaactgaaagaactagatagggatagtgaagaatataaaattataaatgctaagcaaagagtattgaaaataataattaatgcaacctatggctatatgggtttcccaagagcgagatggtattgcatagactgtgctgcggcagtagcagcttggggcaggaaatacattaattatatattaaaaagggccgaagaagaaggattcaaagtaatttatggagataccgattcattattcatttctggggacaaagacaaagtattagaatttttagagaaagtaaataaagaattacccggtaaaatacaattagatttagaagatttctatgttagagggatattcgtaaaaaagaggggtgaacaaaagggggcaaaaaagaaatatgctttattaagcgaacaaggttacataaagctaaggggcttcgaagcagtaagaacagactgggctcccatagttaaagaagtccaaacaaagctattggaaattttgctaaaagaaggtaacatagaaaaagcaagacaatacataaaagaaattattagaaagctaagaaatagagaaataccatgggagaagcttttaattacagaaacgataagaaagcctttagaaaaatacaaagttgaagctcctcatgtggcagcagcaaaaaaatataaaaggttgggctataaagttatgcctggctttagagttagatatttagtggtaggtagcactggaagggtttcagatagaattaaaatagacaaagaagttaggggtaatgaatatgaccccgaatactacatagaaaaacaactattgcctgcagtagagcaaatattagaatctgtaggtattaaagacacattcacaggcaaaaaactaacagatttctttaaaggtaccggcggtggcggtgcaaccgtaaagttcaagtacaaaggcgaagaaaaagaggtagacatctccaagatcaagaaagtatggcgtgtgggcaagatgatctccttcacctacgacgagggcggtggcaagaccggccgtggtgcggtaagcgaaaaggacgcgccgaaggagctgctgcagatgctggagaagcagaaaaagtag
